# Soluble asphaltene oxide: a homogeneous carbocatalyst that promotes synthetic transformations†

**DOI:** 10.1039/d0ra01762k

**Published:** 2020-04-21

**Authors:** Hyosic Jung, Christopher W. Bielawski

**Affiliations:** Center for Multidimensional Carbon Materials (CMCM), Institute for Basic Science (IBS) Ulsan 44919 Republic of Korea bielawski@unist.ac.kr; Department of Chemistry, Ulsan National Institute of Science and Technology (UNIST) Ulsan 44919 Republic of Korea; Department of Energy Engineering, Ulsan National Institute of Science and Technology (UNIST) Ulsan 44919 Republic of Korea

## Abstract

Carbocatalysts, materials which are predominantly composed of carbon and catalyze the synthesis of organic or inorganic compounds, are promising alternatives to metal-based analogues. Even though current carbocatalysts have been successfully employed in a broad range of synthetic transformations, they suffer from a number of drawbacks in part due to their heterogeneous nature. For example, the insolubility of prototypical carbocatalysts, such as graphene oxide (GO), may restrict access to catalytically-active sites in a manner that limits performance and/or challenges optimization. Herein we describe the preparation and utilization of soluble asphaltene oxide (sAO), which is a novel material that is composed of oxidized polycyclic aromatic hydrocarbons and is soluble in a wide range of organic solvents as well as in aqueous media. sAO promotes an array of synthetically useful transformations, including esterifications, cyclizations, multicomponent reactions, and cationic polymerizations. In many cases, sAO was found to exhibit higher catalytic activities than its heterogeneous analogues and was repeatedly and conveniently recycled, features that were attributed to its ability to form homogeneous phases.

## Abstract 2

Soluble carbocatalysts, materials which are predominantly composed of carbon and catalyze the synthesis of organic or inorganic compounds, are promising alternatives to metal-based analogues.

## Graphical abstract

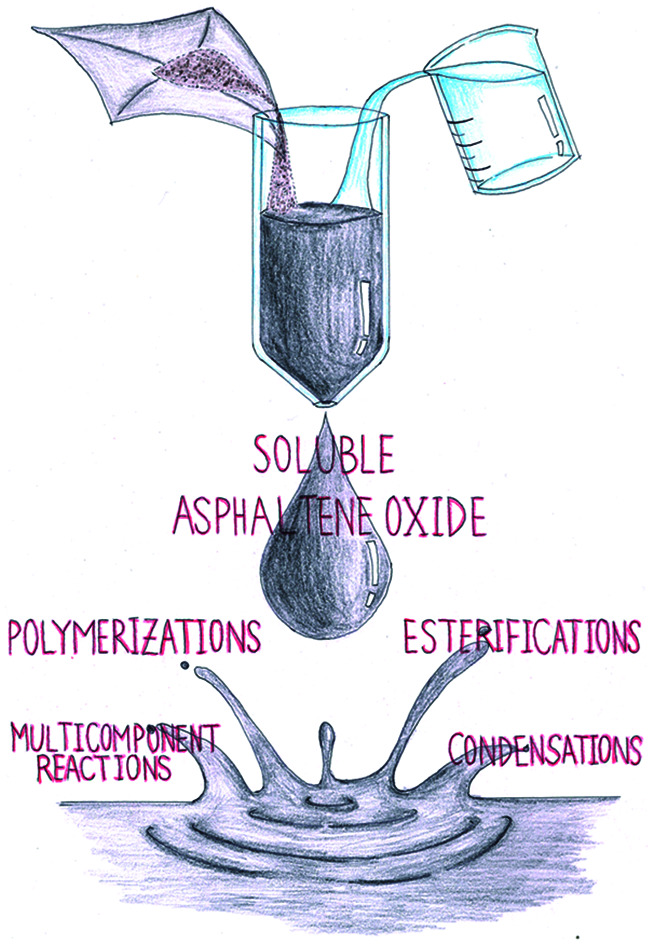

## Introduction

Due to increasing costs, high toxicity, and limited resources, alternatives to transition metal-based catalysts used to promote the preparation of organic and inorganic compounds have been widely studied. Among these, carbocatalysts, which are materials that are composed primarily of carbon, have attracted attention for use in a variety of synthetic applications.^1–3^ The prototypical example of a carbocatalyst is graphene oxide (GO), which was first introduced as a catalyst for the selective oxidation of benzyl alcohol in 2010.^4^ Since the initial disclosure, GO has been reported to catalyze a variety of synthetic reactions, including the oxidations of thiols and sulfides,^5^ aza-Michael additions,^6^ aldol coupling reactions,^7^ C–H activations/coupling reactions,^8–10^ and others.^11^ GO has also been shown to facilitate the polymerization of various monomers through dehydration^12^ or cationic mechanisms.^13^ Despite these advances, GO suffers from a number of fundamental and practical drawbacks. For example, due to the its large particle size, GO can undergo rapid decomposition when exposed to high temperatures, light or microwaves.^14^ Although heterogeneity can facilitate purifications, the catalytically-active sites present on GO may be inaccessible due to its heterogeneous nature and, as such, performance may be intrinsically limited. While enhanced solvent dispersibility has been reported to increase the performance and material quality of GO,^15,16^ a fully soluble derivative has hitherto remained unknown.

We recently reported a new carbocatalyst based on asphaltene.^17^ While the structure of asphaltene is reminiscent of a graphene sheet, it is relatively small.^18^ Subjecting asphaltene to a modified Hummers' method afforded asphaltene oxide (AO), which was found to exhibit catalytic activities similar to that of GO but under a wider range of conditions. Indeed, AO facilitated condensations, C–C cross couplings, etherifications and heterocyclizations, and the use of microwaves were found to promote some of these transformations. Building on these results, we posited that asphaltene may be converted to homogeneous carbocatalyst by enhancing the degree of oxidation. Our supposition was inspired by a previous report,^19^ which showed that exposing asphaltene to 70% nitric acid at elevated temperatures facilitates its removal from crude oil due to an enhanced solubility. Although this procedure was used in oil refinery applications, we hypothesized that the soluble product may function as a homogeneous carbocatalyst. Moreover, the enhanced solubility was expected to promote catalytic function and result in higher activities when compared to heterogeneous counterparts. Herein we report the synthesis and characterization of soluble asphaltene oxide (sAO) and describe the utility of this material in facilitating synthetic transformations, including relatively complex syntheses such as multicomponent reactions and cationic polymerizations.

## Experimental section

### General methodology

All chemicals were purchased from Sigma Aldrich, TCI or Thermo Fisher Scientific Chemicals, and were used as received. Asphaltene (blown asphalt 5–10) was kindly supplied by Korea Petroleum. Unless otherwise noted, all experiments were performed under ambient conditions. NMR spectra were recorded on a Bruker Ascend 400 MHz spectrometer. Chemical shifts are reported in parts per million (*δ*). NMR data were collected in CDCl_3_, DMSO-d_6_ or D_2_O and the residual solvent was referenced to 7.26, 2.50 and 4.79 ppm, respectively. The following abbreviations apply: s, singlet; d, doublet; t, triplet; q, quartet; m, multiplet; br, broad. FT-IR spectra were recorded using KBr pellets on a PerkinElmer Frontier MIR spectrometer. Elemental analyses were performed using a Thermo Scientific Flash 2000 Organic Elemental Analyzer that was calibrated with 2,5-bis(5-*tert*-butyl-benzoxazol-2-yl)thiophene (BBOT). Matrix-assisted laser desorption/ionization (MALDI) spectra were recorded on a Bruker Ultraflex III with 2,5-dihydroxybenzoic acid (DHB) as a matrix. THF solutions of sAO and DHB were combined and then evaporated prior to mass analysis. Polystyrene equivalent molecular weights and polydispersity index (*Đ*) values were measured by gel permeation chromatography (GPC) using a Malvern VISCOTEK GPCmax system. All samples were analyzed using THF as the eluent at a flow rate of 0.8 mL min^−1^. Atomic force microscopy measurements were performed using a Multimode 8 Nanoscope® V (Bruker). Samples were dissolved in THF (1.0 mg mL^−1^) and spin coated onto silica at 3000 rpm for 1 min. Mass spectrometry (MS) data were recorded on a Thermo LCQ Fleet Quadrupole Ion Trap Mass Spectrometer in Atmospheric Pressure Chemical Ionization (APCI) and Electrospray Ionization (ESI) mode (electrospray voltage 3.5 kV). Microwave reactions were conducted using an Anton Paar GmbH-Monowave 300 microwave reactor.

### Separation of asphaltene

Asphaltene (10.0 g) was mixed with *n*-heptane (2.0 L) and then sonicated. The mixture was filtered through a 0.22 μm nylon membrane and washed with *n*-heptane (0.5 L × 3). The filtered asphaltene particles were collected, dried at 80 °C under vacuum for 1 day, and then directly used.

### Preparation of soluble asphaltene oxide (sAO)

A 500 mL flask was charged with asphaltene (4.0 g), nitric acid (0.1 L), and a stir bar. The mixture was refluxed at 120 °C for 24 h. CAUTION: a brown gas, presumably NO_2_, was generated during the reaction. Afterward, the mixture was slowly poured into deionized water (0.5 L). The resulting solution was filtered through a 0.22 μm nylon membrane to remove the unreacted asphaltene particles. The filtrate was collected and dried under vacuum (100 torr) at 100 °C. After removal of the residual water and nitric acid *via* distillation, the remaining solid products were collected and dried under vacuum at 50 °C for 2 days. The final product (2.1 g) was obtained as a brown powder.

## Results and discussion

**Fig. 1:**
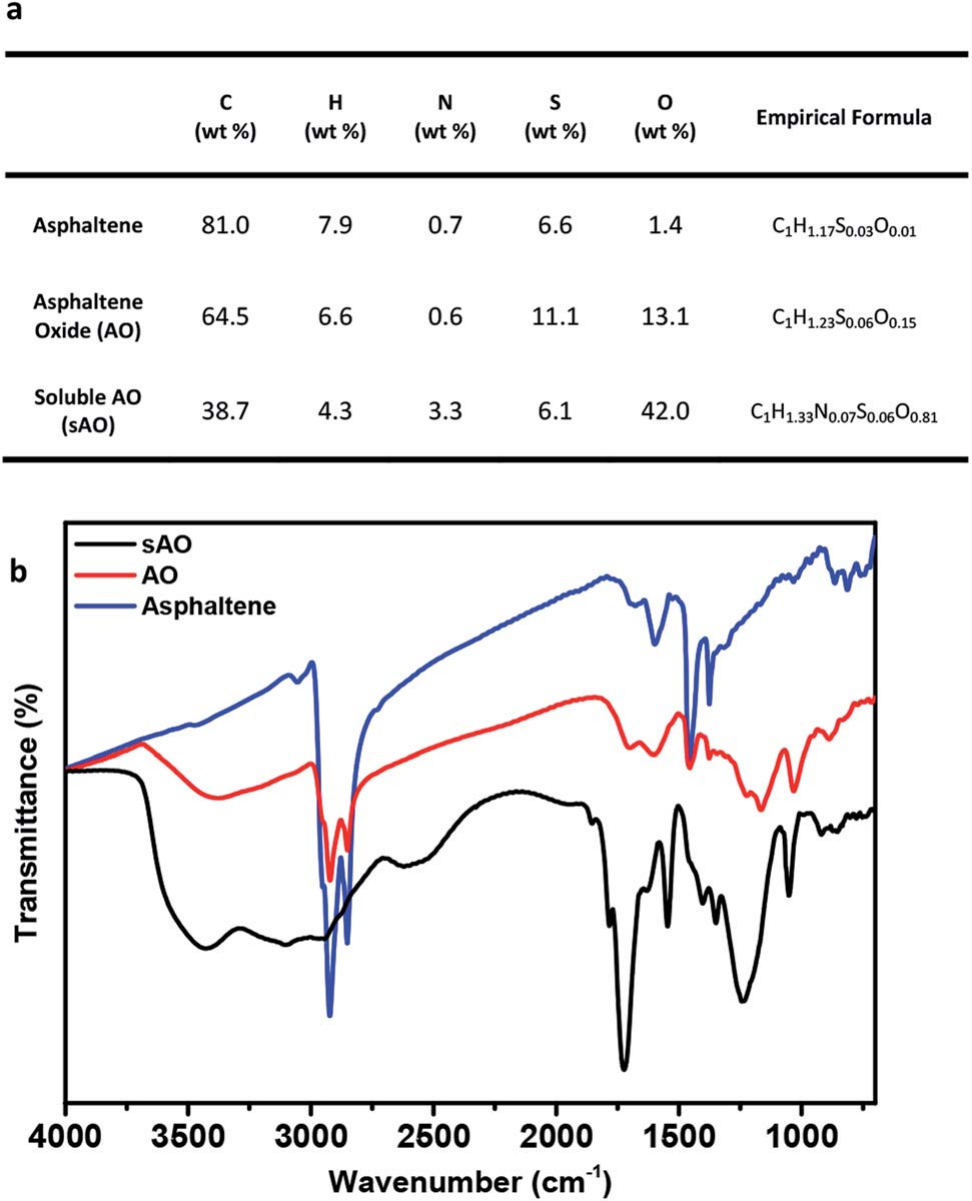
Summary of characterization data. (a) Elemental analysis data recorded for asphaltene, AO and sAO. (b) FT-IR spectra recorded for asphaltene (blue line), asphaltene oxide (red line) and sAO (black line).

Asphaltene was first washed with *n*-heptane to remove the residual oil contaminants and then subjected to a modified version of a previously reported oxidation method.^19^ Briefly, asphaltene was refluxed in 60% nitric acid at 120 °C for 24 h, and then diluted with deionized water. After filtering the diluted solution to remove the insoluble particulates, the filtrate was dried by heating to 100 °C under low pressure and then placed in a vacuum oven that was set to 50 °C for two days. The product obtained from this procedure was a brown powder and found to be soluble in methanol (up to 200 mg mL^−1^), ethanol (140 mg mL^−1^), THF (200 mg mL^−1^), DMSO (80 mg mL^−1^), DMF (80 mg mL^−1^), acetone (200 mg mL^−1^) and water (100 mg mL^−1^) (see Fig. S1†). Compared to asphaltene oxide (AO), which was synthesized using a modified Hummers' method (conditions: KMnO_4_, H_2_SO_4_, and H_2_O_2_), sAO contained a relatively high oxygen content (13.1 wt%, C/O ratio ∼ 6.67 *vs.* 42.0 wt%, C/O ratio ∼ 1.23, respectively), as determined by elemental analysis (see Fig. 1a). The FT-IR spectrum recorded for the material featured a strong, broad hydroxyl signal (*ν*_O–H_ = 3500–2800 cm^−1^), a strong carbonyl signal (*ν*_C=O_ = 1720 cm^−1^), and epoxide signals (*ν*_C–O_ = 1240 cm^−1^ and 1050 cm^−1^), and resembled a spectrum recorded for AO (see Fig. 1b). The pH of a solution of sAO in water (1.0 mg mL^−1^) was measured to be 2.7; for comparison, the pH of an AO suspension was measured to be 3.5 under similar conditions.

**Scheme 1:**
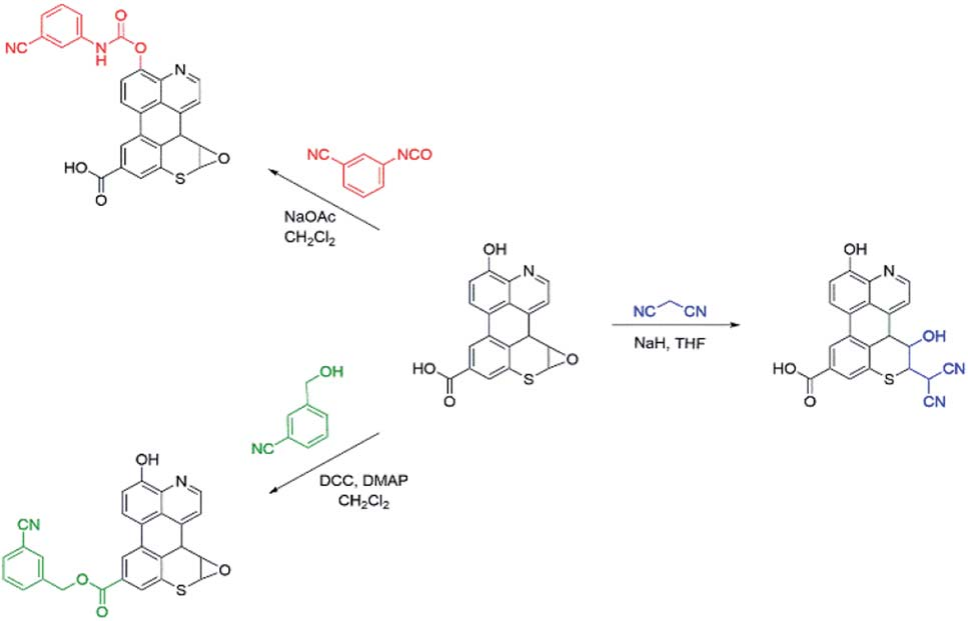
Chemical tests that were used to quantify the functional groups displayed on sAO. The starting material shown in the centre represents a truncated form sAO and features pendant carboxylic acid, hydroxy, and epoxide groups. Using the reagents shown, the carboxylic acid groups were converted to esters, the hydroxyl groups were converted to carbamates, and epoxide groups were ring-opened. In all cases, the corresponding products feature nitrile groups (CN) that could be quantified using IR spectroscopy as well as elemental analysis.

As summarized in Scheme 1, a series of chemical tests were deployed to elucidate the structure of sAO and to quantify the oxygen-containing functional groups decorated on the material. The reagents utilized were designed to preferentially react with carboxylic acid, hydroxy or epoxide groups and contained nitriles to facilitate characterization *via* IR spectroscopy and elemental analysis (see Fig. S2 and Table S1,† respectively). Treatment of sAO with 3-cyanobenzyl alcohol under Steglich esterification conditions^20^ afforded the corresponding ester, as determined in part by a bathochromic shift in the salient *ν*_C=O_ signal (from 1720 cm^−1^ to 1630 cm^−1^) and the appearance of a new signal which was assigned to a nitrile group (*ν*_C≡N_ = 2230 cm^−1^). Introducing sAO to 3-cyanophenyl isocyanate resulted in a reduction in the intensity of the signal assigned to the hydroxyl group (*ν*_O–H_ = 3320 cm^−1^) and was accompanied by the formation of strong signals that were assigned to the nitrile (*ν*_C≡N_ = 2230 cm^−1^), amido (*ν*_N–H_ = 3090 cm^−1^) and carbamyl (*ν*_C=O_ = 1590 cm^−1^) groups of the expected product. Finally, using methodology that was previously reported to form C–C bonds *via* the ring-opening of the epoxide groups on GO,^21^ sAO was treated with malononitrile under basic conditions. The disappearance of signals attributed to the epoxide groups (*ν*_C–O_ = 1230 cm^−1^) in the starting material was observed along with the formation a new *ν*_C≡N_ at 2180 cm^−1^. Elemental analysis of the products of the aforementioned reactions enabled quantification of the respective nitrogen contents and thus the corresponding functional groups: carboxylic acid (5.1 × 10^−3^ mol g^−1^), hydroxy (5.6 × 10^−3^ mol g^−1^) and epoxide (7.5 × 10^−3^ mol g^−1^). The concentrations of these groups were measured to be significantly higher than those measured for AO and GO (see Table S2†), which may rationalize the relatively high solubility and high catalytic activity (*vide infra*) exhibited by sAO.

The relatively high solubility of sAO facilitated additional characterization of the material. A ^1^H NMR spectrum recorded for the material exhibited broad signals at *δ* 8.62–7.41 ppm and 2.31–1.00 ppm in D_2_O which were assigned to aromatic and alkyl chain protons, respectively (see Fig. S3†). Similarly, ^13^C NMR signals attributed to carbonyl groups (*δ* 179.0–176.9 ppm), aromatic groups (*δ* 134.4–129.0 ppm) and alkyl chains (*δ* 40.4–16.1 ppm) were also recorded (see Fig. S4†). Matrix assisted laser desorption/ionization time of flight (MALDI-TOF) mass spectrometry revealed that the molecular weight of sAO was in the range of 500–600 Da (see Fig. S5†). Presumably due to its relatively high oxygen content, the material was found to aggregate in the solid-state and formed particles with an average size of *ca.* 10 nm, as determined by atomic force microscopy (AFM) (see Fig. S6†).

**Scheme 2:**
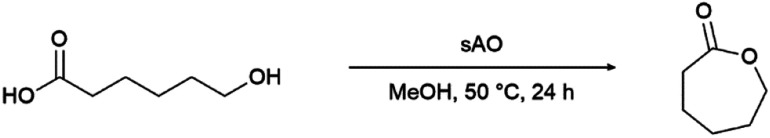
Intramolecular condensation of 6-hydroxycaproic acid as catalyzed by sAO.

Since sAO was found to be acidic, initial efforts toward using sAO as a carbocatalyst began with esterification chemistry. Although inorganic acids such as hydrochloric acid, sulfuric acid and phosphoric acid are commonly used to catalyze such transformations,^22^ these liquid reagents are toxic and volatile. For comparison, sAO is a solid powder that is relatively straightforward to handle. As summarized in Table 1, sAO successfully promoted the condensation of a wide range of carboxylic acids and alcohols. Esterifications involving carboxylic acids and methyl alcohol quantitatively afforded the corresponding methyl esters while other alcohols were converted to their respective esters in yields of up to 96%. An intramolecular esterification was also explored. As shown in Scheme 2, 6-hydroxycaproic acid (1.0 mmol) was successfully converted to the expected product (ε-caprolactone) in quantitative yield using sAO (conditions: [6-hydroxycaproic acid]_0_ = 1.0 M; 50 mg sAO).

**Table 1:** A summary of esterifications that were promoted with sAO^a^

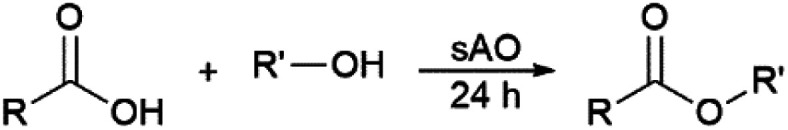				
Entry	Carboxylic acid	Alcohol	Temp. (°C)	Conversion^b^ (%)
1	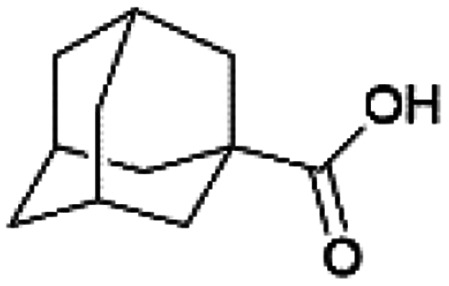	MeOH	50	>99
2	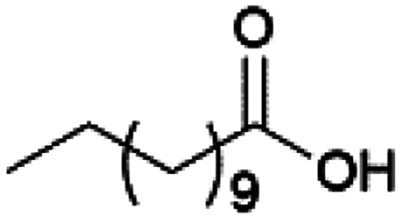	MeOH	50	>99
3	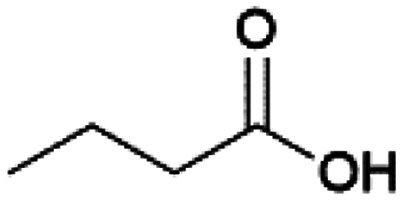	MeOH	50	>99
4	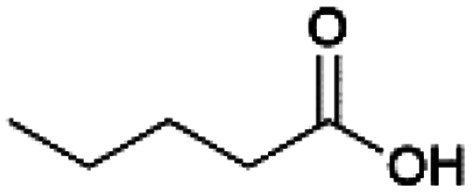	MeOH	50	>99
5	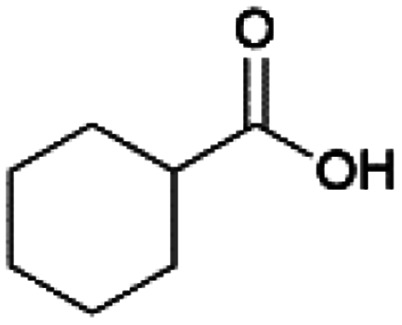	MeOH	50	>99
6	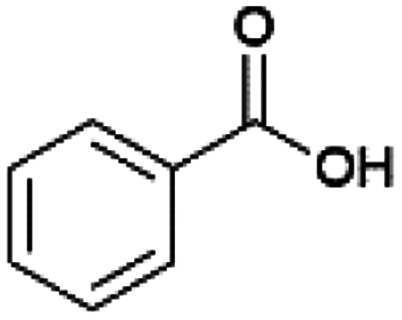	MeOH	50	>99
7	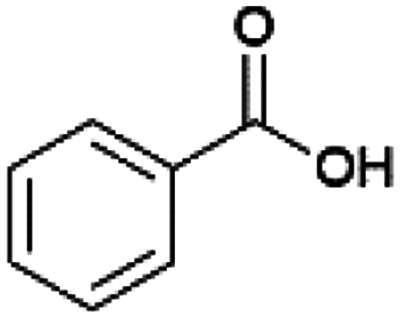	EtOH	70	91
8	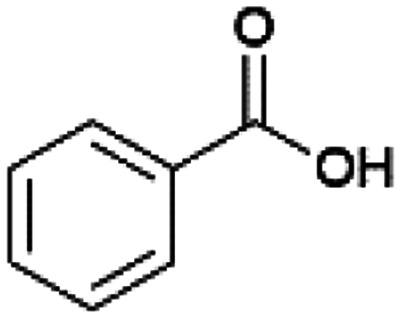	*n*-BuOH	70	96
9	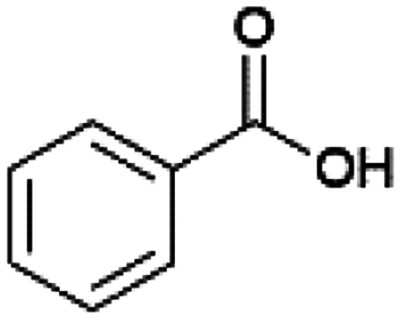	IPA	85	85

a Unless otherwise noted, all reactions were performed using 1.0 mmol of carboxylic acid, 1.0 mL of alcohol, and 50 mg of catalyst.

b Conversion after 24 h as calculated by gas chromatography against a standard (anisole).

We previously showed that asphaltene-based carbocatalysts feature an advantage over their graphene analogues in that they can be utilized in microwave-assisted chemistry.^17^ Building on these results, we focused on employing sAO in microwave-assisted Fischer indole syntheses. Indole derivatives have been employed in numerous biological and pharmaceutical applications and the demand for these compounds continues to grow.^23^ As shown in Table 2, sAO successfully facilitated the condensation of phenylhydrazine and cyclohexanone to afford the corresponding indole product in aqueous media. Similarly, other ketones were converted to their expected indoles when irradiated with microwaves in the presence of sAO.

**Table 2:** A summary of microwave assisted Fischer indole syntheses^a^

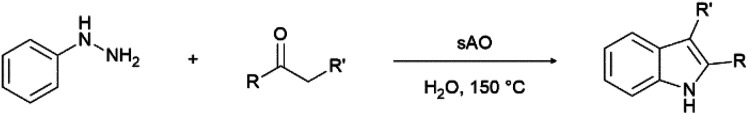					
Entry	Ketone	Product	Catalyst (mg)	Time (min)	Yield^b^ (%)
1	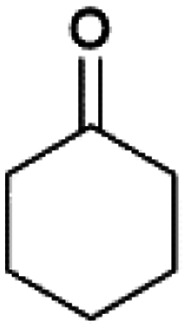	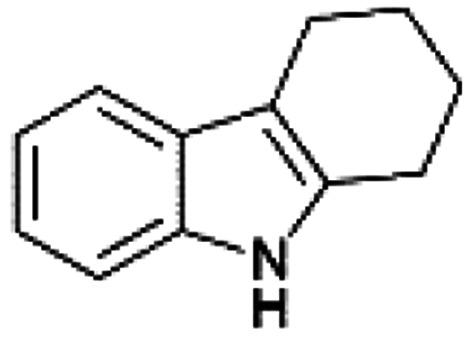	100	30	30
2	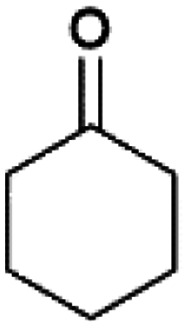	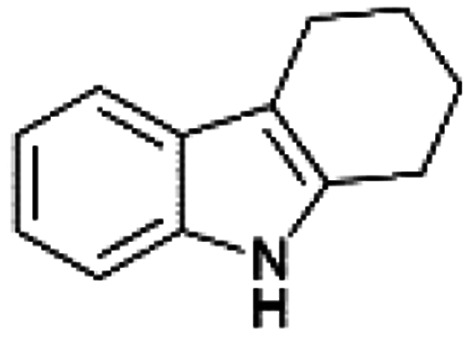	100	60	60
3	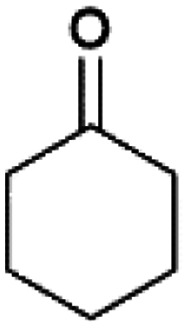	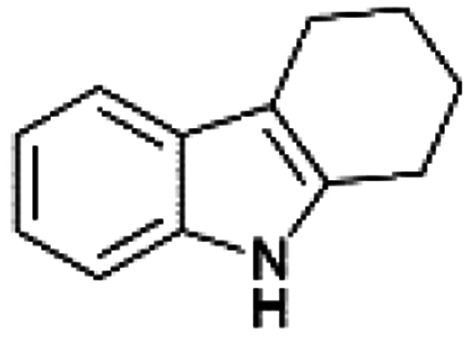	100	90	62
4	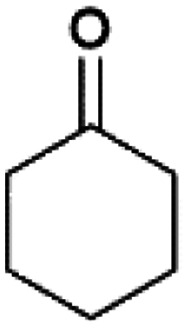	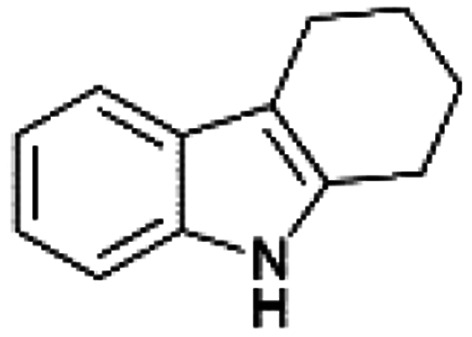	200	60	61
5	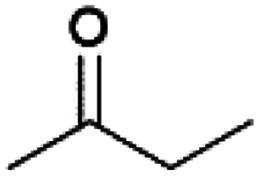	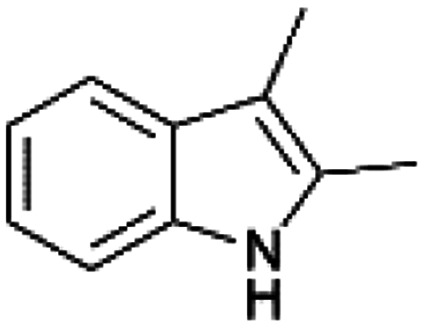	100	60	26
6	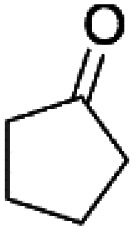	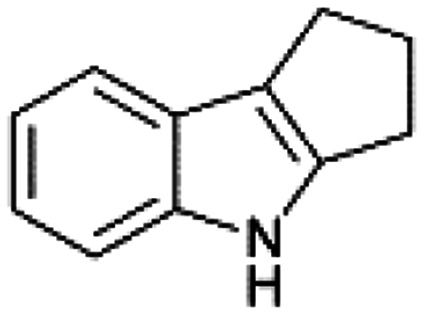	100	60	37
7	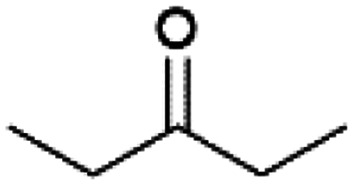	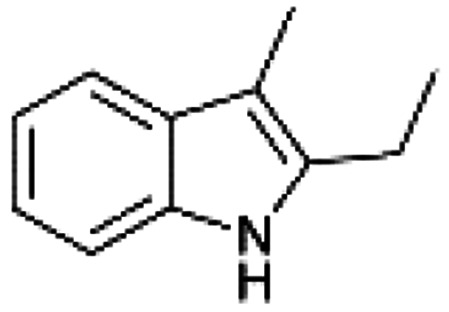	100	60	45

a Unless otherwise noted, all reactions were performed using 1.2 mmol of phenylhydrazine, 1.0 mmol of cyclohexanone, 2.0 mL of water as solvent and the indicated quantity of catalyst loading at 150 °C in a microwave reactor.

b Isolated yield after purification using column chromatography.

A hallmark of many carbocatalysts is that they can be readily recycled. Since sAO is soluble in water, separation of the catalyst from various product mixtures was conveniently accomplished *via* extraction. To quantify the recyclability of sAO, an aqueous solution of the catalyst was collected after a Fischer indole synthesis and re-used without further purification. The starting reagents (phenylhydrazine and cyclohexanone) were added to a solution of the catalyst in water ([sAO]_0_ = 50 mg mL^−1^) and then irradiated with microwaves for 1 h at 150 °C. After extraction of the corresponding indole products with dichloromethane, the aqueous phase containing the catalyst was collected, introduced to a new bolus of starting reagents and irradiated again. Using this methodology, the catalyst solution was successfully and repeatedly used over multiple cycles to facilitate condensations with minimal changes catalytic activity. For example, an indole product was obtained in a 52% yield using a catalyst solution that was subjected to five (5) successive cycles; for comparison, a yield of 59% was obtained after the first cycle (see Table S3†).

Considering that the catalytic activity displayed by sAO appeared to be relatively broad, we hypothesized that the material may also facilitate multicomponent reactions (MCRs). The Biginelli reaction is a typical example of a MCR and has been used to obtain dihydropyrimidinone (DHPM) and other valuable heterocyclic products.^24^ Since DHPM derivatives are known to be effective calcium channel blockers, they have found widespread utility in therapeutic and pharmacological applications.^25^ As summarized in Table 3, a series of MCRs were conducted with a benzaldehyde derivative, ethyl acetoacetate and urea at various catalyst loadings (10–50 wt%), temperatures (50–100 °C) and reaction periods (5–30 min). While DHPM products were isolated from each experiment, optimal results were obtained when the reaction mixture was irradiated with microwaves for 5 min at 100 °C (see entry 5). For comparison, an analogous reaction that was performed with AO afforded a 33% yield of product under otherwise identical conditions. Other benzaldehydes, including those containing electron-rich or electron-poor functional groups, were also utilized and afforded the expected products in good yields (see Table S4†).

**Table 3:** A summary of microwave-assisted Biginelli reactions^a^

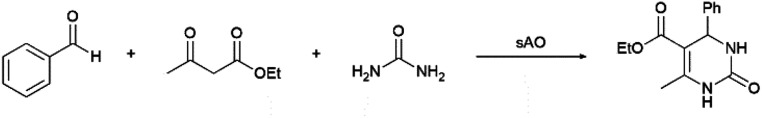				
Entry	Cat. loading (wt%)	Temp. (°C)	Time (min)	Yield^b^ (%)
1	10	75	5	13
2	25	75	5	21
3	50	75	5	53
4	50	50	5	7
5	50	100	5	75
6	50	100	15	74
7	50	100	30	75

a Unless otherwise noted, all reactions were performed with benzaldehyde (1.0 mmol), ethyl acetoacetate (1.0 mmol) and urea (1.5 mmol) using the indicated catalyst loading.

b Isolated yield after purification using column chromatography.

Finally, we explored the ability of sAO to catalyze cationic polymerizations. Vinyl carbazole and vinyl ethers are ideal monomers for such chemistry since the nitrogen or oxygen heteroatoms effectively stabilize propagating carbocations. As summarized in Table 4, the aforementioned monomers were found to undergo polymerization when subjected to AO (1 wt%) in THF at −20 °C, and the corresponding polymers were isolated *via* filtration after pouring the corresponding reaction mixtures into excess methanol to induce precipitation. Poly(*n*-butyl vinyl ether) was obtained in quantitative yield, and featured a number average molecular weight (*M*_n_) of 9.8 kDa and a polydispersity index (*Đ*) of 1.3 as determined by gel permeation chromatography. Similarly, *N*-vinyl carbazole was quantitatively converted to its corresponding polymer (*M*_n_ = 11.1 kDa). Although the latter exhibited a *Đ* value of 5.1, broad polydispersity indices are often observed in cationic polymerizations due to the high reactivity of the cation during the propagation step.^26^ Reduced distributions may be achieved through further optimization (see Table S5†). While GO has also used to promote cationic polymerizations,^13^ sAO afforded polymers in higher yields, with better control, and within shorter periods of time.

**Table 4:** A summary of cationic polymerization that were performed with sAO^a^

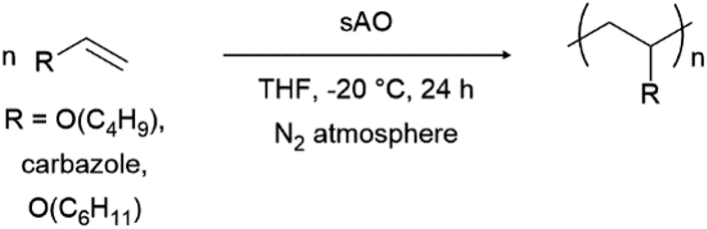					
Entry	R	Conc. (mol L^−1^)	Yield^b^ (%)	*M*_n_ c (kDa)	*Đ* c
1	–O(C_4_H_9_)	3	99	9.8	1.3
2	–Cbz	1	99	11.1	5.1
3	–O(C_6_H_11_)	3	52	4.5	2.0

a The reactions were performed by treating a solution of the monomer in THF with 1 wt% of sAO at −20 °C for 24 h under an atmosphere of N_2_.

b Isolated yield after collection of the precipitate that formed upon pouring the reaction mixture into excess methanol.

c The number-average molecular weight (*M*_n_) and polydispersity index (*Đ*) values were determined by GPC and are reported as their polystyrene equivalents. Cbz = carbazole.

## Conclusions

In summary, a homogeneous carbocatalyst was prepared through the oxidation of asphaltene. To the best of our knowledge, this is the first report of a soluble carbocatalyst that facilitates a broad range of synthetic transformations. Indeed, sAO effectively promoted esterifications, inter- and intramolecular condensations, multicomponent reactions and cationic polymerizations, and was successfully used in microwave reactors. Since sAO is soluble in water, it was found to be straightforward to separate and recycle *via* extraction. Moreover, due to its ability to form homogeneous phases, sAO exhibited higher catalytic activities than its heterogeneous counterparts (*e.g.*, GO or AO). In a broader perspective, asphaltene-based catalysts can be expected to expand the realization of metal-free catalysts for facilitating synthetic reactions under heterogeneous and, now, homogeneous conditions, and thus potentially usher further improvements in catalytic activity as well as selectively.

## Conflicts of interest

There are no conflicts to declare.

## Supplementary Material

RA-010-D0RA01762K-s001
