# Supplementary material for: Soluble asphaltene oxide: a homogeneous carbocatalyst that promotes synthetic transformations
Source: RSC Adv. 2020 Apr 21;10(26):15598–603. doi: 10.1039/d0ra01762k (PMC9052364; doi:10.1039/d0ra01762k)
Supplement: RA-010-D0RA01762K-s001 [file RA-010-D0RA01762K-s001.pdf]

Electronic Supplementary Material (ESI) for RSC Advances.
This journal is © The Royal Society of Chemistry 2020

Supporting Information

Soluble Asphaltene Oxide: A Homogeneous Carbocatalyst That Promotes Synthetic Transformations

Hyosic Jung[a, b] *and Christopher W. Bielawski*[a, b, c,*]

[a] Center for Multidimensional Carbon Materials (CMCM), Institute for Basic Science (IBS), Ulsan 44919, Republic of Korea

[b] Department of Chemistry, Ulsan National Institute of Science and Technology (UNIST), Ulsan 44919, Republic of Korea

[c] Department of Energy Engineering, Ulsan National Institute of Science and Technology (UNIST), Ulsan 44919, Republic of Korea

Identification and quantification of the carboxylic acids on sAO.[1] A 100 mL Schlenk flask was charged with 50.0 mg of sAO, 10.0 mL of CH_2Cl_2 and a stir bar under an atmosphere of N_2. To the flask were added 3-cyanobenzyl alcohol (200.0 mg, 1.5 mmol) and 4-(dimethylamino)pyridine (DMAP; 10.0 mg, 0.1 mmol), and the resulting mixture was cooled to 0 °C in an ice bath. In a separate flask, N,N'-dicyclohexylcarbodiimide (DCC; 155.0 mg, 0.8 mmol) was dissolved in 5.0 mL of CH_2Cl_2. The DCC solution was added dropwise to a flask containing sAO at 0 °C. The combined mixture was stirred at 40 °C for 24 h and then filtered. The isolated solids were washed with excess CH_2Cl_2 and then collected with THF. Vacuum drying for 1 day facilitated the removal of the residual THF and afforded a yellow powder. The final product was termed 'modified sAO-1' (m-sAO-1) and characterized using FT-IR spectroscopy as well as elemental analysis. A new nitrile stretching frequency was observed at 2230 cm^{-1} in the FT-IR spectrum recorded for the product and the carbonyl signal measured in the sAO starting material (1720 cm^{-1}) shifted to 1630 cm^{-1}.

Identification and quantification of the hydroxyl groups on sAO. A 100 mL round bottom flask was charged with 50.0 mg of sAO, 10.0 mL of CH_2Cl_2 and a stir bar. Sodium acetate (123.0 mg, 1.5 mmol) and 3-cyanophenyl isocyanate (216.2 mg, 1.5 mmol) were subsequently added to the flask, and the resulting mixture was then stirred at 25 °C for 24 h. After filtration, the isolated solids were washed with excess CH_2Cl_2 and then collected with THF. Vacuum drying for 1 day facilitated the removal of the residual THF and afforded a yellow powder. The final product was termed 'modified sAO-2' (m-sAO-2) and characterized using FT-IR spectroscopy as well as elemental analysis. The FT-IR spectrum recorded for the product revealed an attenuated hydroxyl stretching frequency (3320 cm^{-1}) as well as strong absorptions that were attributed to the nitrile (2230 cm^{-1}) and carbamoyl (1590 cm^{-1}) groups from the expected condensation product.

Identification and quantification of the epoxide groups on sAO.[2] A 50 mL flask was charged with sodium hydride (82.0 mg, 3.4 mmol), malononitrile (220.0 mg, 3.3 mmol) and 30.0 mL of THF under an atmosphere of N_2. The mixture was stirred for 30 min. A separate 100 mL Schlenk flask was charged with 40.0 mg of sAO, 10.0 mL of THF and a stir bar under an atmosphere of N_2. After cooling both mixtures on an ice bath, the malononitrile solution was added dropwise to the sAO solution at 0 °C. The resulting mixture was then heated at 60 °C for 24 h. The solvent was subsequently evaporated, and the resulting solids were washed with excess CH_2Cl_2 to remove the residual malononitrile. The solids were then collected with THF. Vacuum drying for 1 day facilitated the removal of the residual THF and afforded a yellow powder. The final product was termed 'modified sAO-3' (m-sAO-3) and characterized using FT-IR spectroscopy as well as elemental analysis. The FT-IR spectrum recorded for the product revealed the presence of a signal that was assigned to a nitrile group (2180 cm^{-1}). In addition, the intensity of the signal assigned to the epoxy groups (1230 cm^{-1}) was relatively reduced upon when compared to the spectrum recorded for the sAO starting material.

General esterification procedure. A Teflon-lined 8 mL vial was charged with 1.0 mmol of a carboxylic acid, 1.0 mL of alcohol to serve both as a reagent and solvent, sAO (50.0 mg), and a stir bar. The vial was sealed under ambient atmosphere with Teflon tape. The vial was heated to a predetermined temperature for 24 h. The resulting mixture was cooled to room temperature and anisole (1.0 mmol), which was used as an external standard, was then added. To calculate the conversion of the reaction, aliquots were periodically collected and analyzed by GC and GC-MS. The final products were not isolated.

Representative Fischer indole synthesis procedure. A Teflon-capped 10 mL microwave vial was charged with phenylhydrazine (129.8 mg, 1.2 mmol), cyclohexanone (98.2 mg, 1.0 mmol), sAO (100.0 mg), water (2.0 mL) and a stir bar. The vial was then microwaved

at 150 °C for 1 h. The resulting mixture was cooled to room temperature and the product was extracted with CH_2Cl_2 and water. The organic phase was collected and dried over $MgSO_4$. The crude product was purified using column chromatography (ethyl acetate : *n*-hexane, 1:9 v/v as eluent) to afford the desired product. Spectroscopic and analytical data were in accord with literature values.[3] ^{1}H NMR (400 MHz, $CDCl_3$): δ 7.64 (s, 1H), 7.46-7.44 (d, 1H), 7.29-7.27 (d, 1H), 7.14-7.07 (m, 2H), 2.74-2.70 (m, 4H), 1.91-1.87 (m, 4H).

Representative Biginelli reaction procedure. A Teflon capped 10 mL microwave vial was charged with benzaldehyde (1.0 mmol), ethyl acetoacetate (1.0 mmol), urea (1.5 mmol), sAO (50.0 mg) and a stir bar. The vial was then microwaved at 100 °C for 5 min. The resulting mixture was cooled to room temperature and then extracted with ethyl acetate and water. The organic phase was collected and dried over $MgSO_4$. The crude product was purified using column chromatography (ethyl acetate : *n*-hexane, 3:7 v/v as eluent). Spectroscopic and analytical data were in accord with literature values.[4] ^{1}H NMR (400 MHz, DMSO-d_6): δ 9.18 (s, 1H), 7.72 (s, 1H), 7.33-7.22 (m, 5H), 5.13-5.12 (d, 1H), 3.99-3.94 (q, 2H), 2.24 (s, 3H), 1.09-1.02 (t, 3H).

General cationic polymerization procedure. A 20 mL Schlenk tube was charged with monomer (1.0 g), sAO (10.0 mg), THF (2.64 – 5.17 mL) and a stir bar at -20 °C under an atmosphere of N_2. After stirring the reaction mixture at -20 °C for 24 h, it was concentrated and poured into excess methanol. The precipitated solid was collected, dried under vacuum, and then characterized.

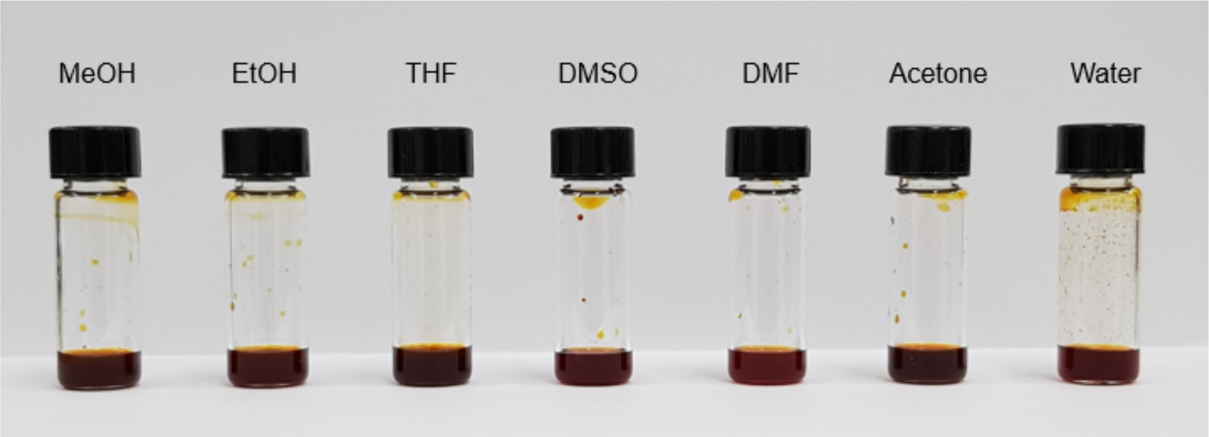

Fig. S1 Photograph of vials which contain sAO dissolved in various solvents (indicated). The concentrations of sAO are as follows: methanol, 200 mg/mL; ethanol, 140 mg/mL; THF, 200 mg/mL; DMSO, 80 mg/mL; DMF, 80 mg/mL; acetone, 200 mg/mL; and water, 100 mg/mL.

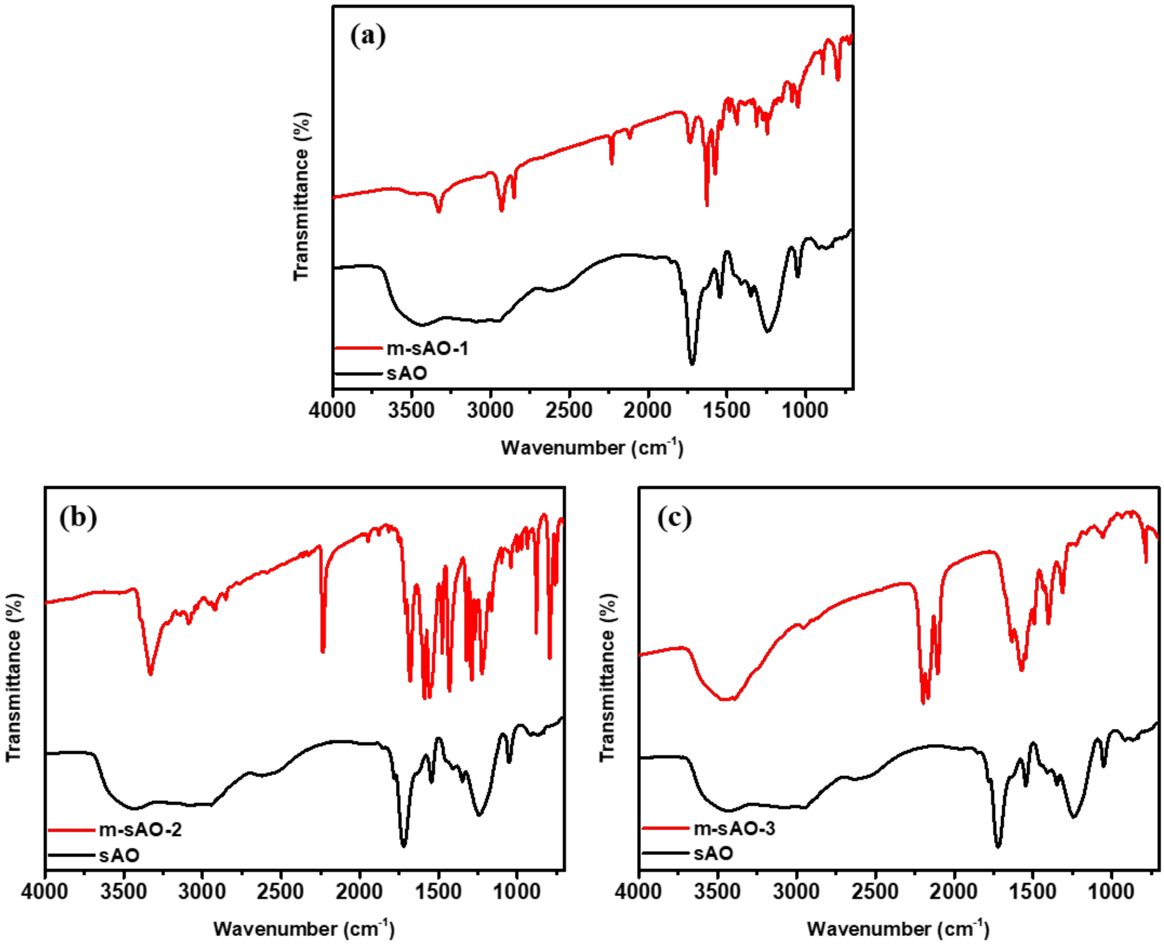

Fig. S2 FT-IR spectra recorded for (a) m-sAO-1 as prepared by treating sAO with 3-cyanobenzyl alcohol under Steglich esterification conditions, (b) m-sAO-2 as prepared by treating sAO with 3-cyanophenyl isocyanate, and (d) m-sAO-3 as prepared by treating sAO with malononitrile under basic conditions.

Table S1 Summary of elemental data recorded for AO and its modified derivatives.

Entry	Carbon (wt%)	Hydrogen (wt%)	Nitrogen (wt%)	Sulfur (wt%)	Oxygen (wt%)	Empirical Formula
AO	40.1	4.4	3.1	6.6	39.5	$C_{1.00}H_{1.30}N_{0.07}S_{0.06}O_{0.73}$
m-sAO-1	68.1	6.8	10.3	0	11.7	$C_{1.00}H_{1.20}N_{0.13}O_{0.13}$
m-sAO-2	66.3	4.4	18.8	0	7.5	$C_{1.00}H_{0.80}N_{0.24}O_{0.08}$
m-sAO-3	43.0	3.4	24.2	0	12.5	$C_{1.00}H_{0.95}N_{0.48}O_{0.22}$

Table S2 Summary of functional group quantification data recorded for sAO, AO and GO[a]

Functional Group	sAO (mol/g)	AO (mol/g)	GO (mol/g)
COOH	$> 5.1 \times 10^{-3}$	$> 2.3 \times 10^{-3}$	$> 3.6 \times 10^{-4}$
OH	$> 5.6 \times 10^{-3}$	$> 1.4 \times 10^{-3}$	$> 1.1 \times 10^{-4}$
epoxide	$> 7.5 \times 10^{-3}$	$> 5.4 \times 10^{-4}$	$> 1.2 \times 10^{-3}$

[a] The calculations were based on the elemental analysis data (see Table S1).

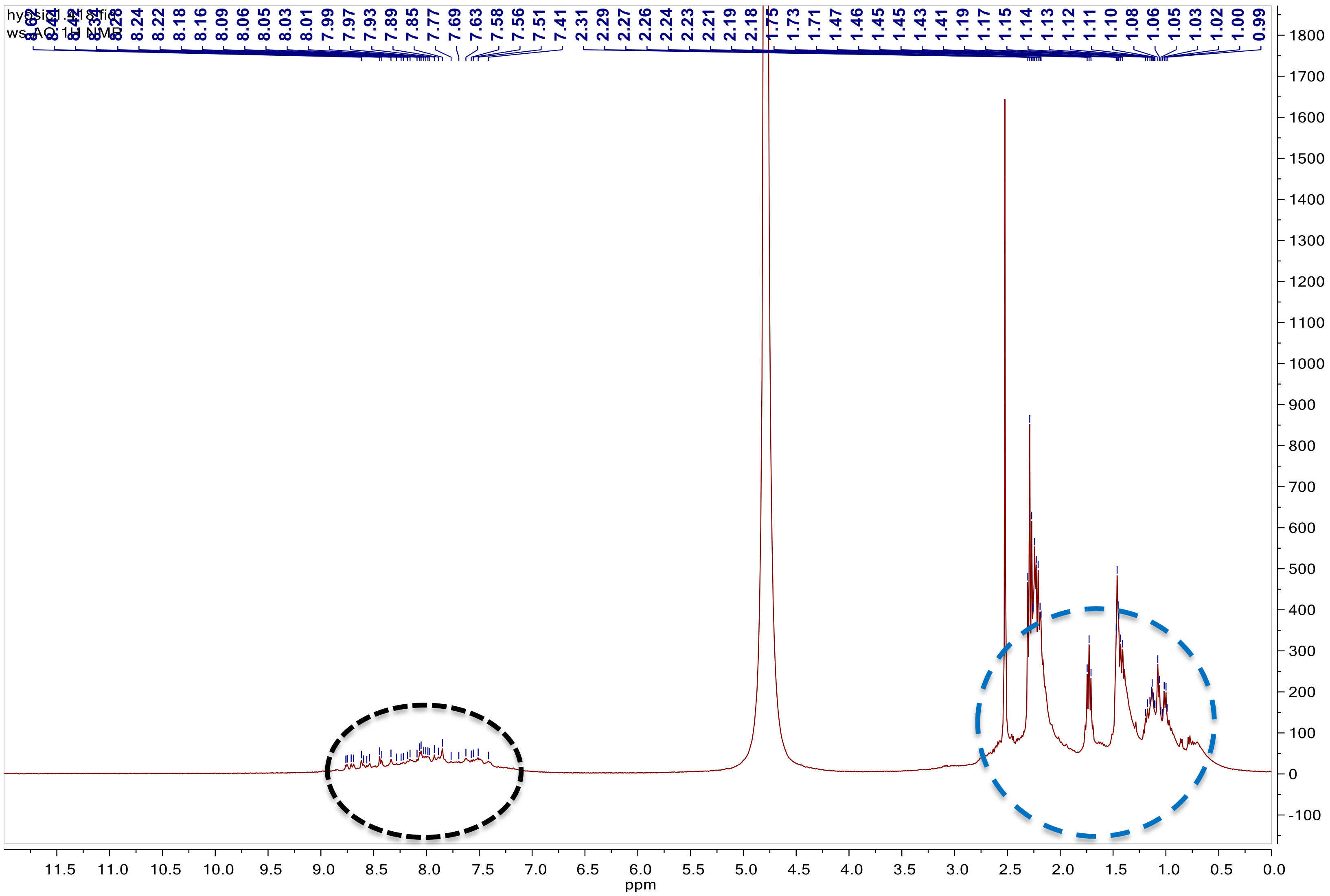

Fig. S3 ^{1}H NMR spectrum recorded for sAO (D_2O).

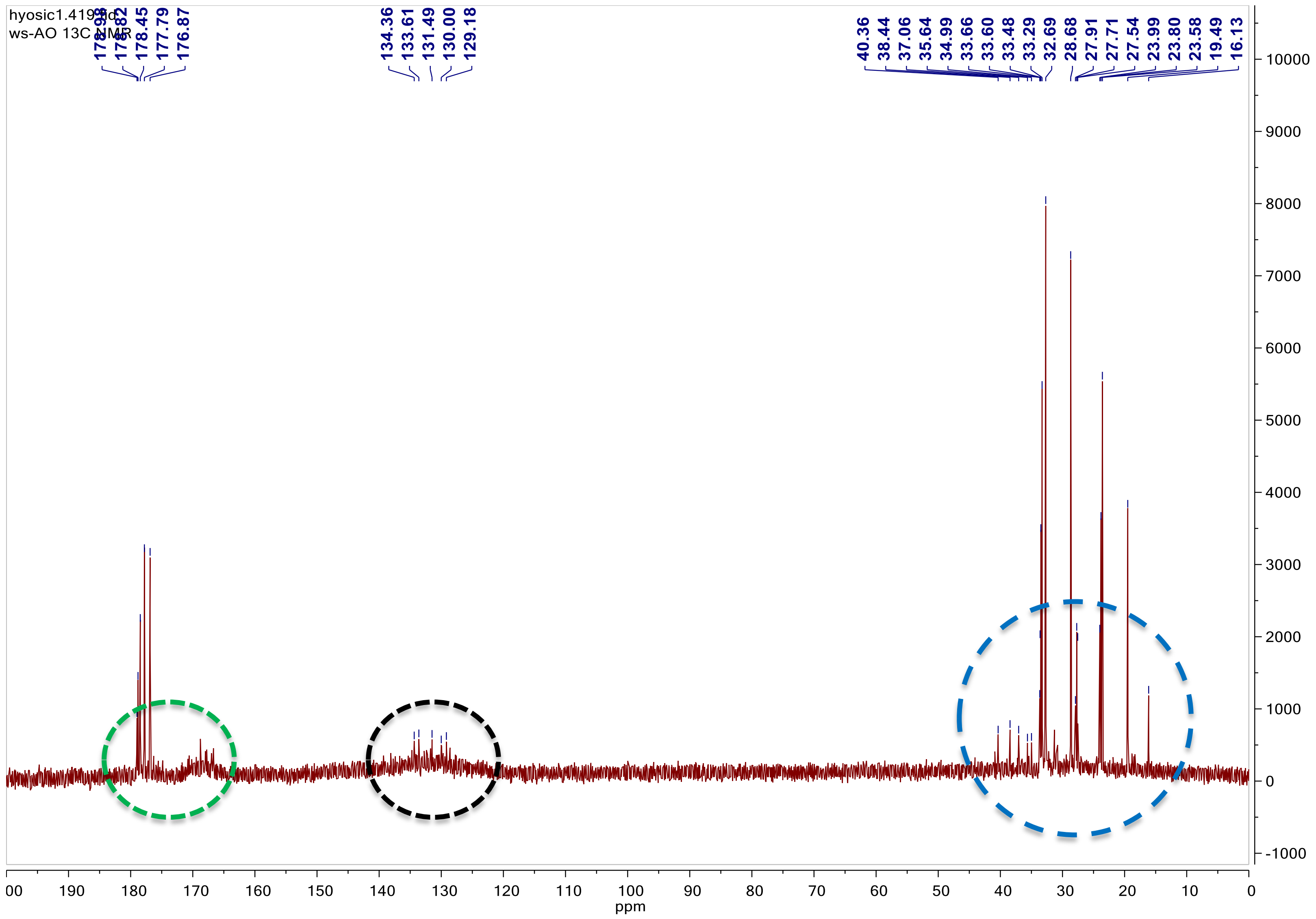

Fig. S4 ^{13}C NMR spectrum recorded for sAO (D_2O).

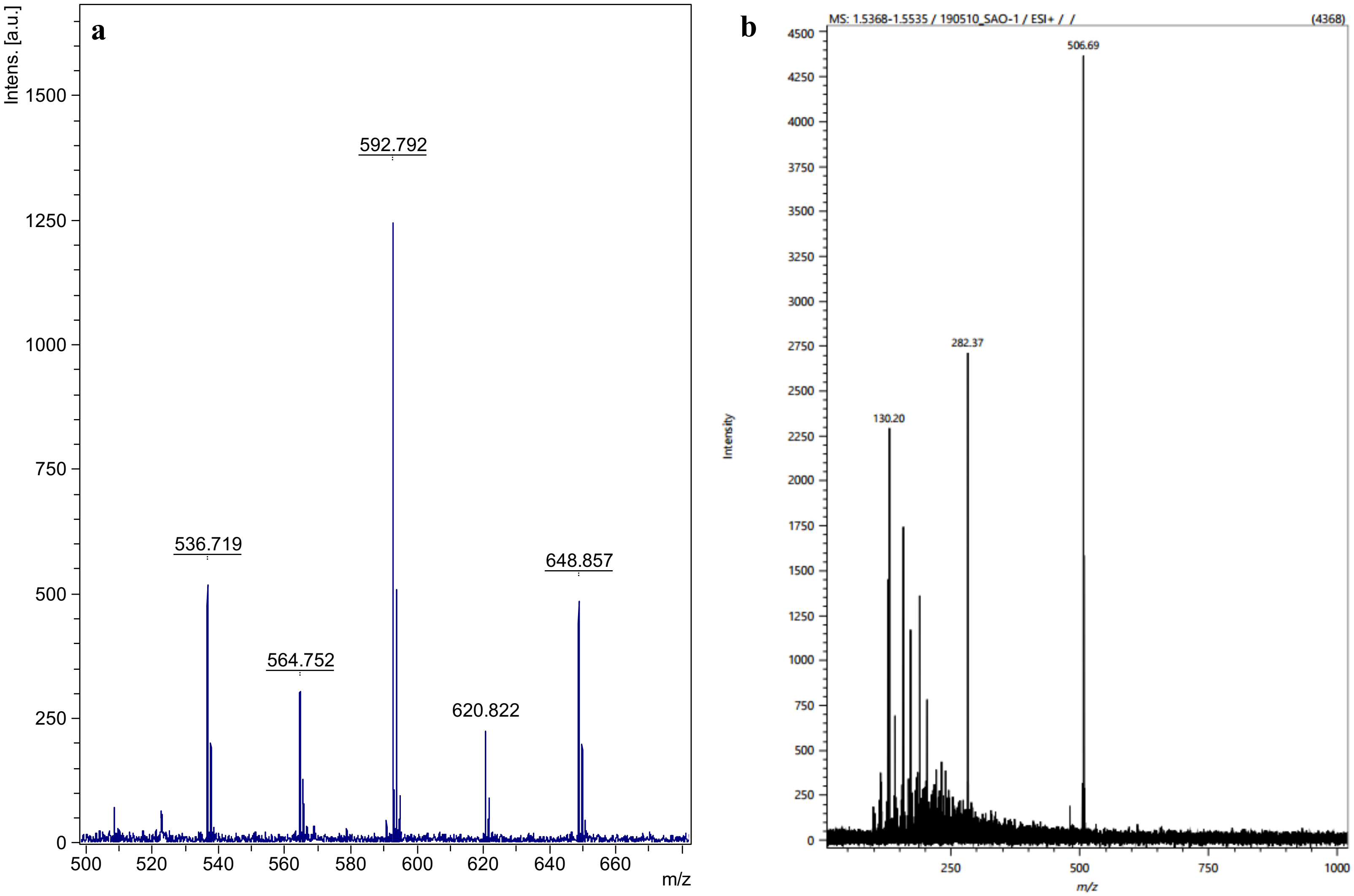

Fig. S5 (a) MALDI-TOF data recorded for sAO. (b) ESI MS data recorded for sAO in the positive mode.

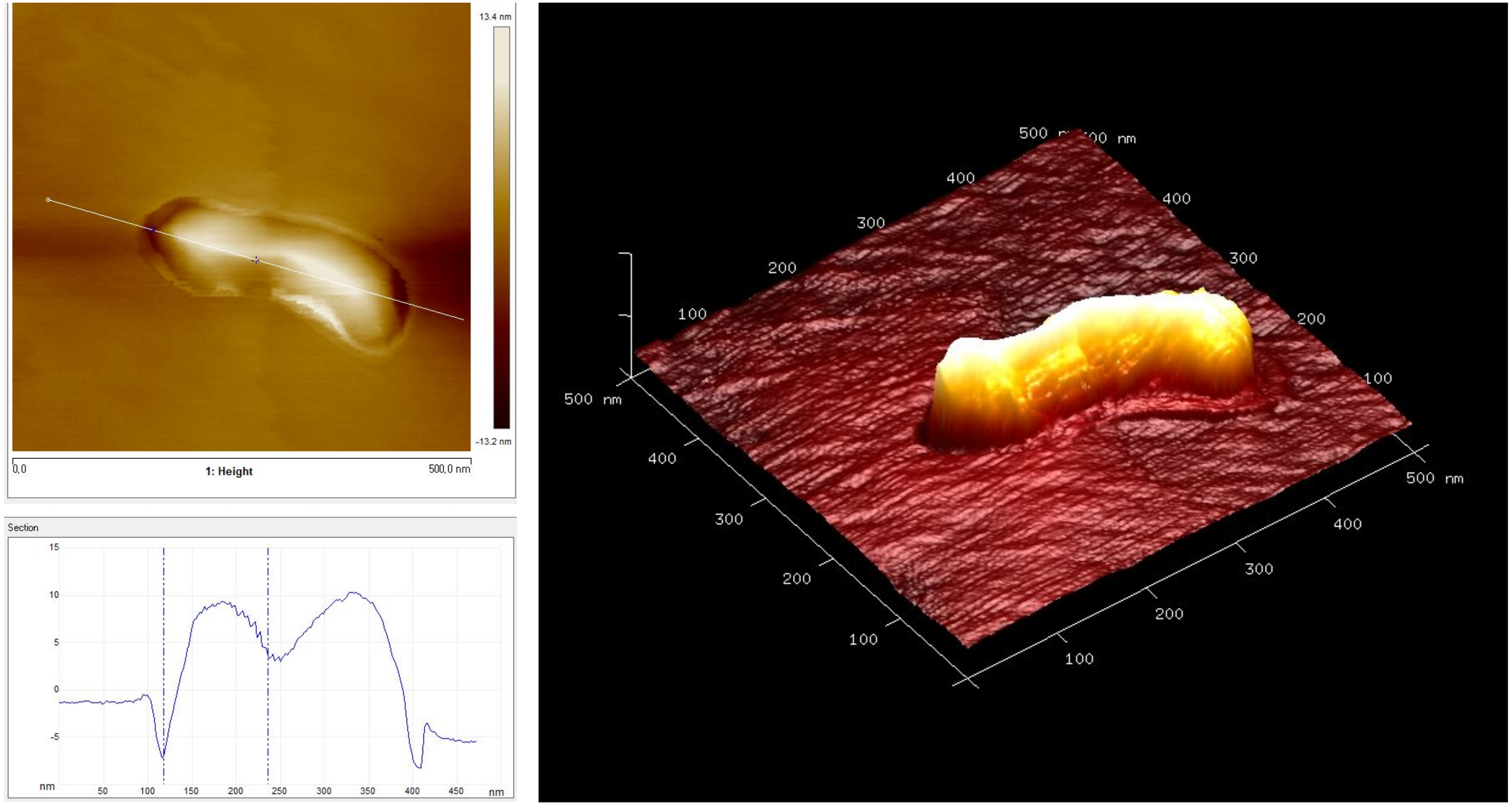

Fig. S6 AFM images recorded for sAO on silica. Conditions used to create films: 1 mg/mL in THF; spinning rate = 3000 rpm for 1 min. Images were collected in the tapping mode using non-contact tips with a spring constant of 6 N m^{-1} and tip radii of ≤ 8 nm (Bruker RTESP-150).

Table S3 A summary of a series of experiments designed to assess the recyclability of sAO[a]

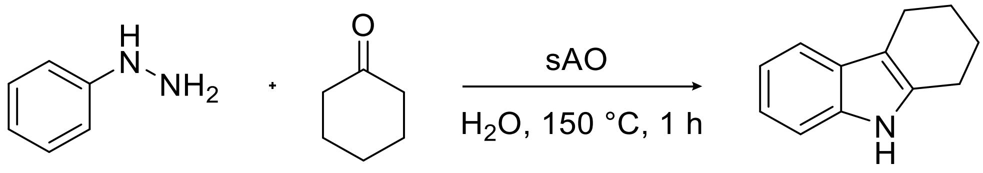

Entry	Yield[b] (%)
1st cycle	59
2nd cycle	53
3rd cycle	57
4th cycle	53
5th cycle	52

[a] All reactions were performed using phenylhydrazine (1.2 mmol), cyclohexanone (1.0 mmol), sAO (100 mg) and water (2.0 mL) at 150 °C for 1 h in a microwave reactor. [b] Isolated yield after purification using column chromatography.

Table S4 A summary of the scope of substrates that was explored for the Biginelli reactions described in the main text[a]

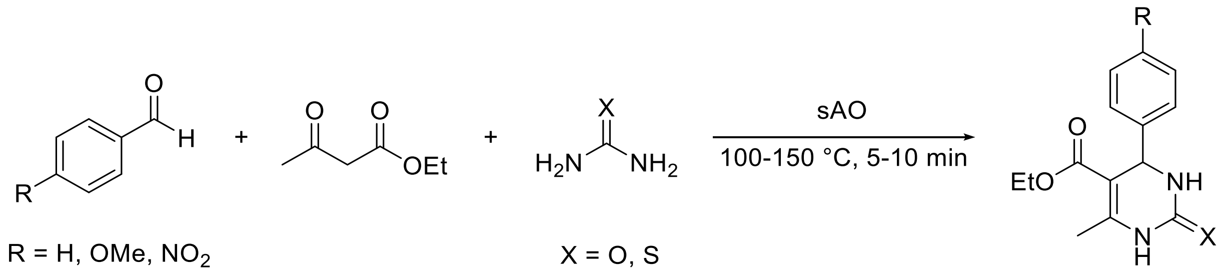

Entry	R	X	Time (min)	Yield[b] (%)
1	H	O	5	75
2	OMe	O	5	72
3	NO_2	O	5	69
4	H	S	10	61
5	OMe	S	10	69
6[c]	NO_2	S	10	64

[a] All reactions were performed with aldehyde (1.0 mmol), ethyl acetoacetate (1.0 mmol) and urea (1.5 mmol) using sAO (50.0 mg) at 100 °C in a microwave reactor. [b] Isolated yield after purification using column chromatography. [c] The reaction was performed with aldehyde (1.0 mmol), ethyl acetoacetate (5.0 mmol), thiourea (1.5 mmol) and sAO (50.0 mg) at 150 °C for 10 min.

Table S5 Optimization of the polymerization of N-vinylcarbazole[a]

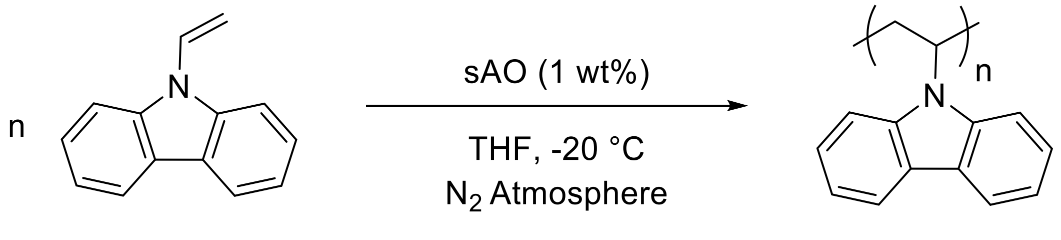

Entry	Time (h)	Yield[b] (%)	M_n[c] (Da)	*Đ*[c]
1	24	99	11.1	5.1
2	4	99	9.5	5.0
3	2	96	9.1	4.1
4	1	96	8.8	4.4
5	0.167 (10 min)	94	9.0	4.3

[a] The reactions were performed by treating a solution of N-vinylcarbazole in THF (1 mol/L) with 1 wt% of sAO at -20 °C under an atmosphere of N_2. [b] Isolated yield after collection of the precipitate formed upon pouring the reaction mixture into excess methanol. [c] The number-average molecular weight (M_n) and polydispersity index (*Đ*) were determined by GPC and are reported as their polystyrene equivalents.

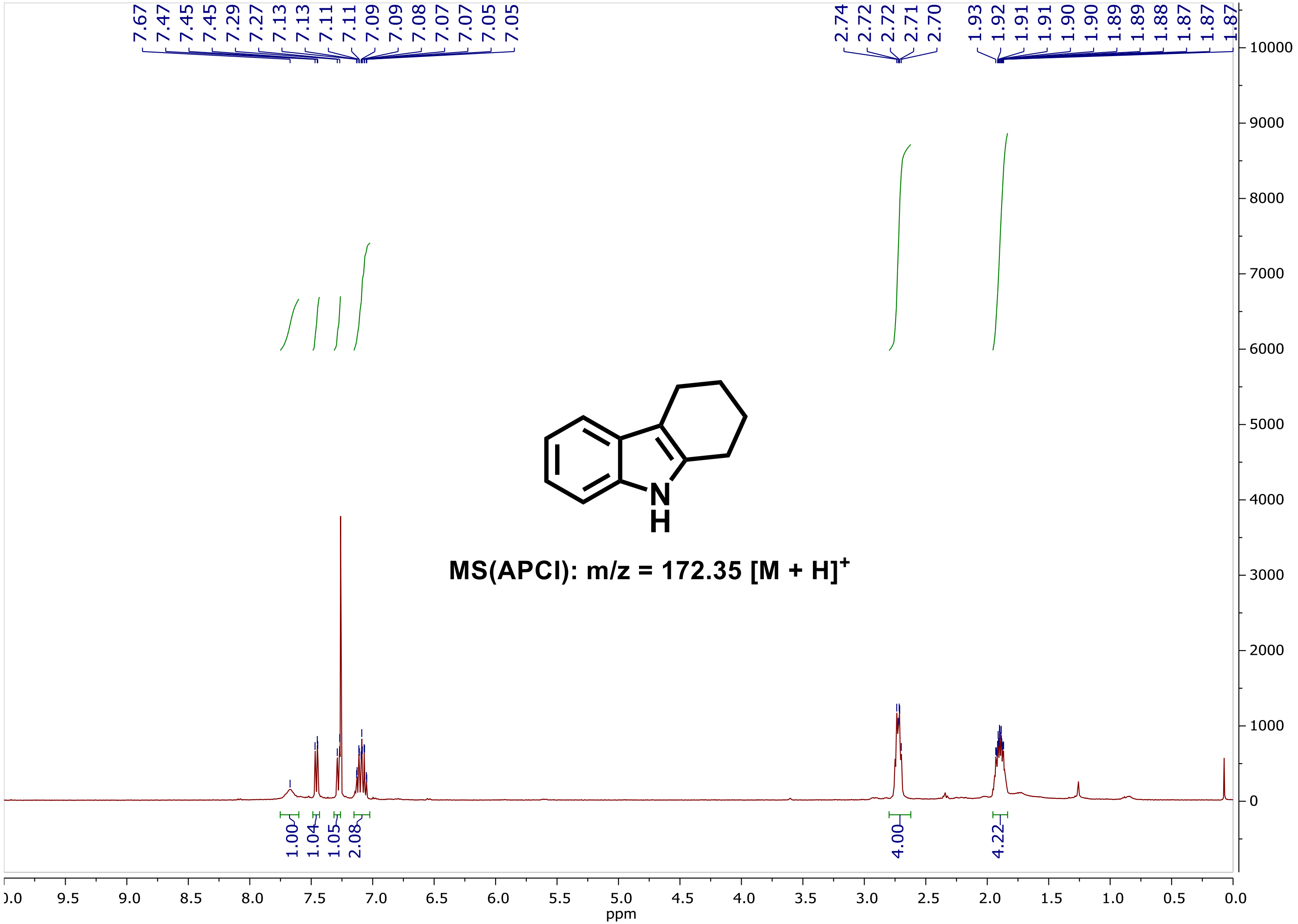

Fig. S7 ^{1}H NMR spectrum recorded for 1,2,3,4-tetrahydrocarbazole ($CDCl_3$). Data were in accord with literature values.[3]

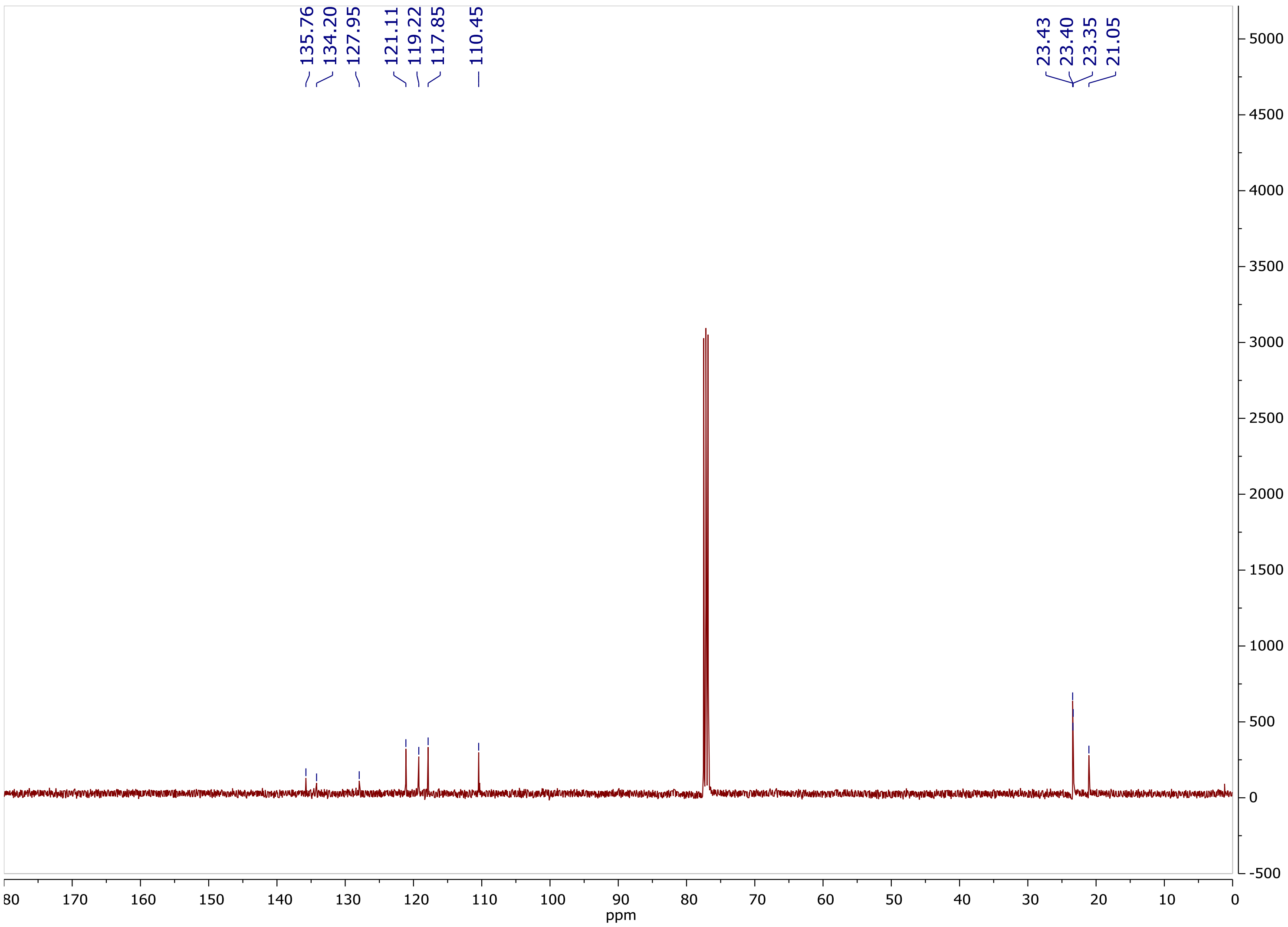

Fig. S8 ^{13}C NMR spectrum recorded for 1,2,3,4-tetrahydrocarbazole ($CDCl_3$).

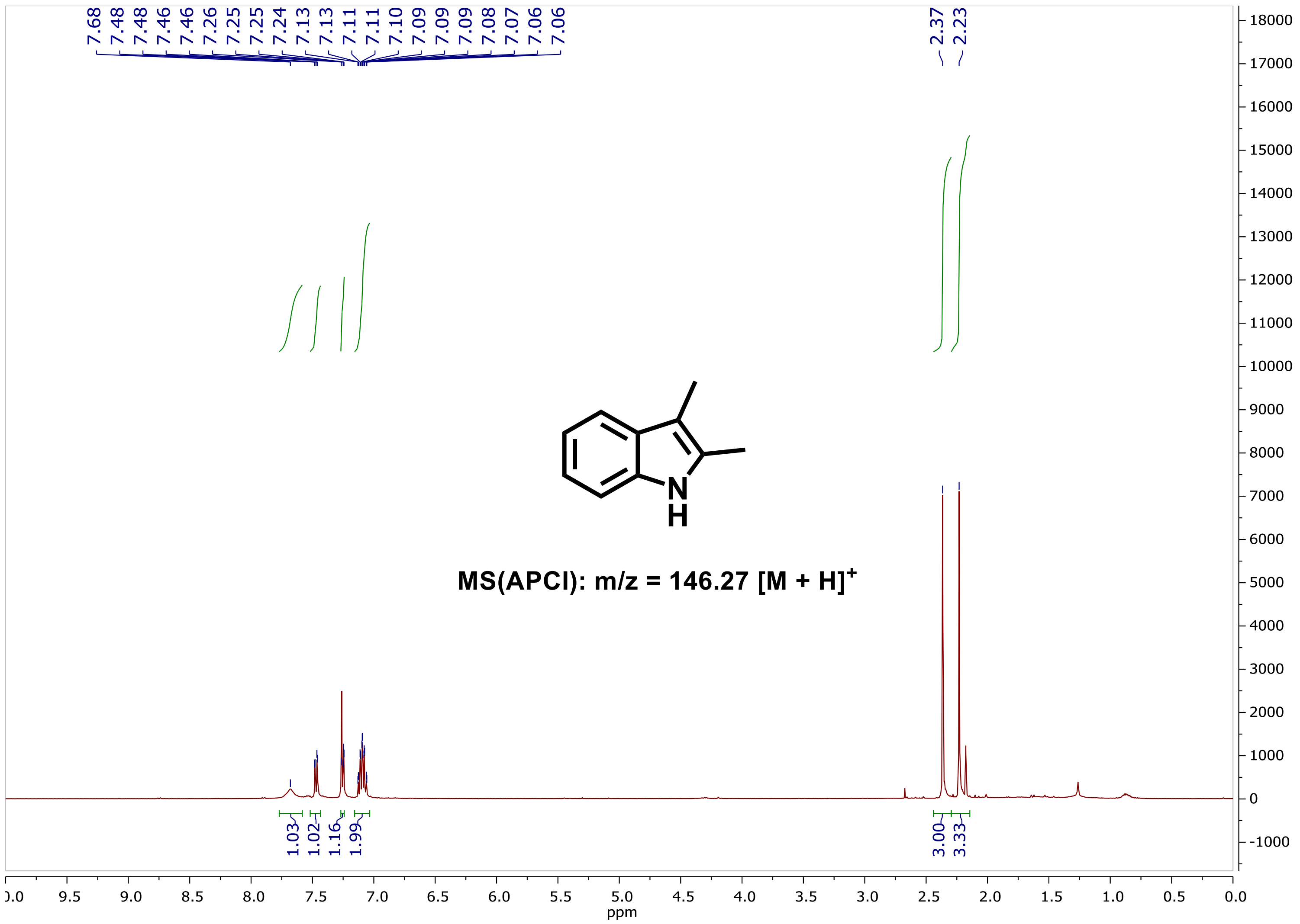

Fig. S9 ^{1}H NMR spectrum recorded for the product described in Table 2, Entry 5 ($CDCl_3$). Data were in accord with literature values.[3]

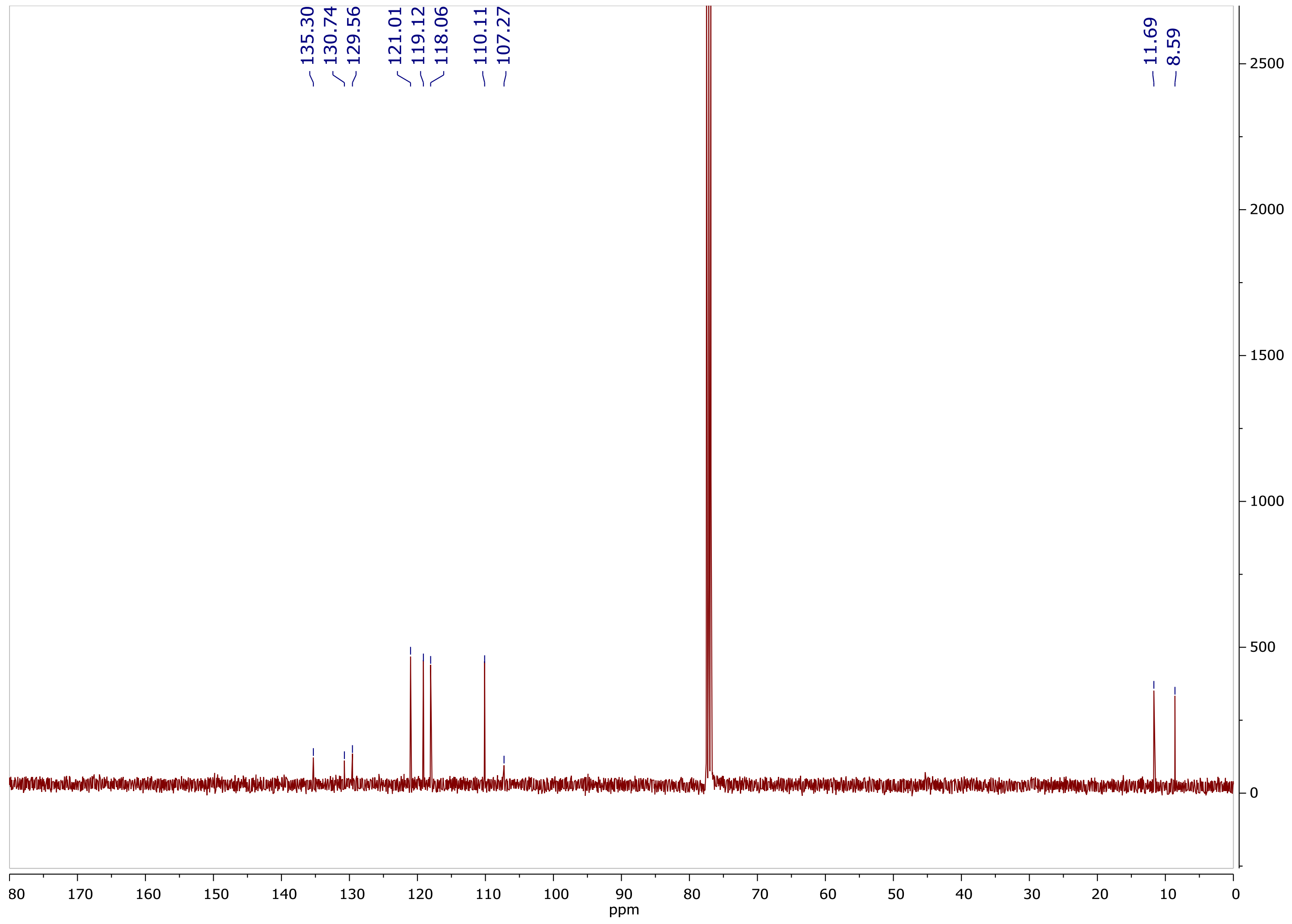

Fig. S10 ^{13}C NMR spectrum recorded for the product described in Table 2, Entry 5 ($CDCl_3$).

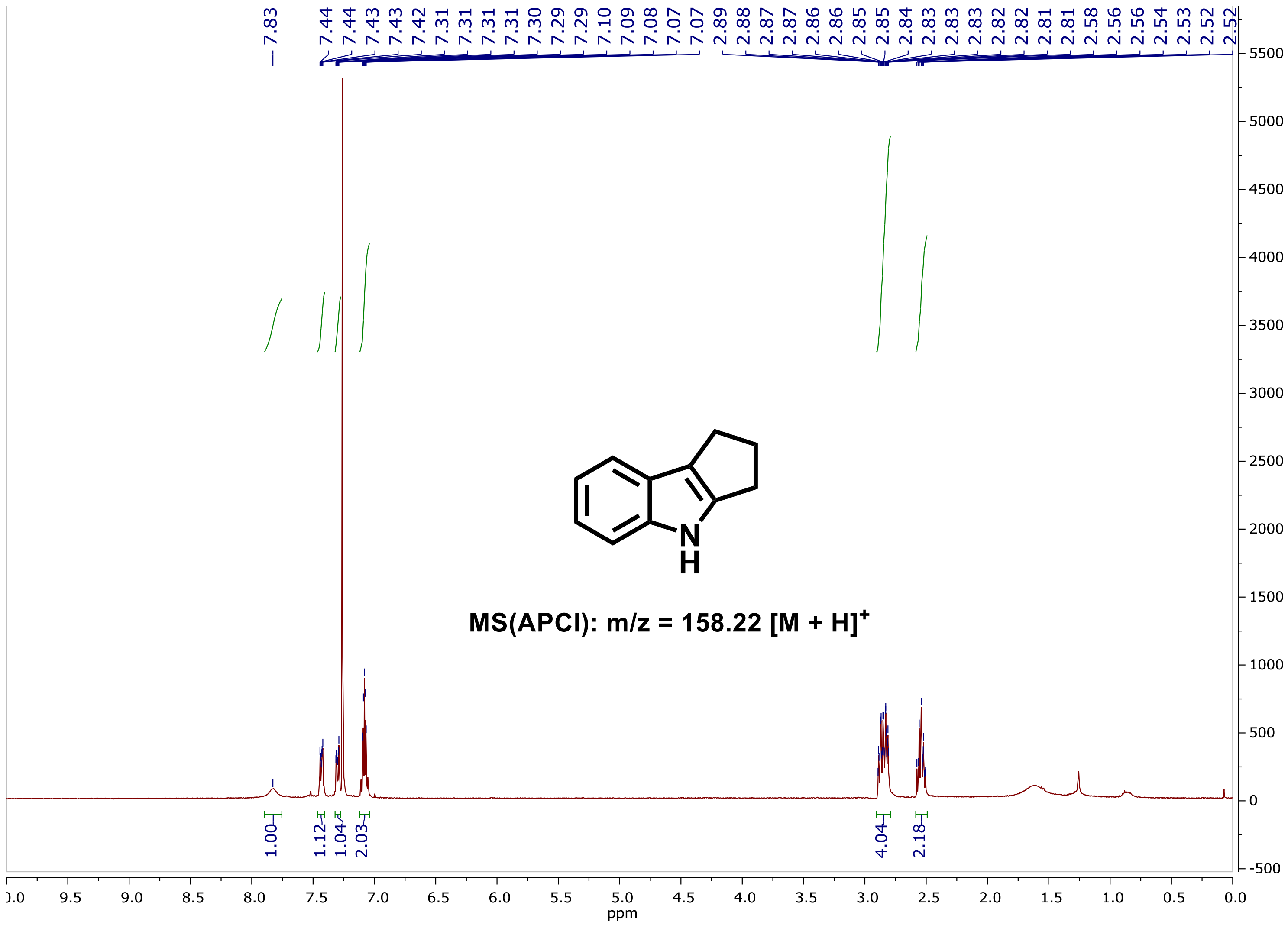

Fig. S11 ^{1}H NMR spectrum recorded for the product described in Table 2, Entry 6 ($CDCl_3$). Data were in accord with literature values.[3]

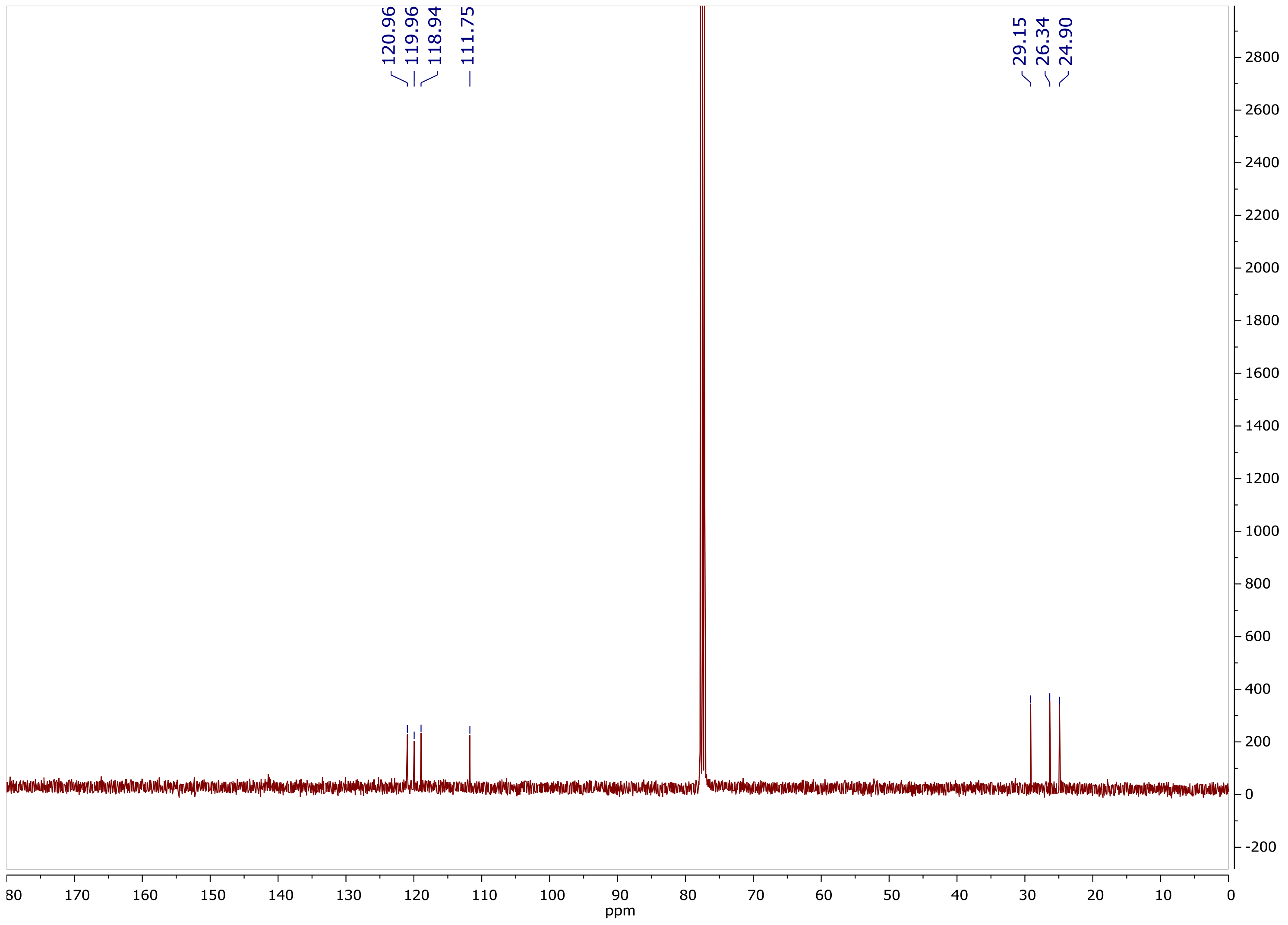

Fig. S12 ^{13}C NMR spectrum recorded for the product described in Table 2, Entry 6 ($CDCl_3$).

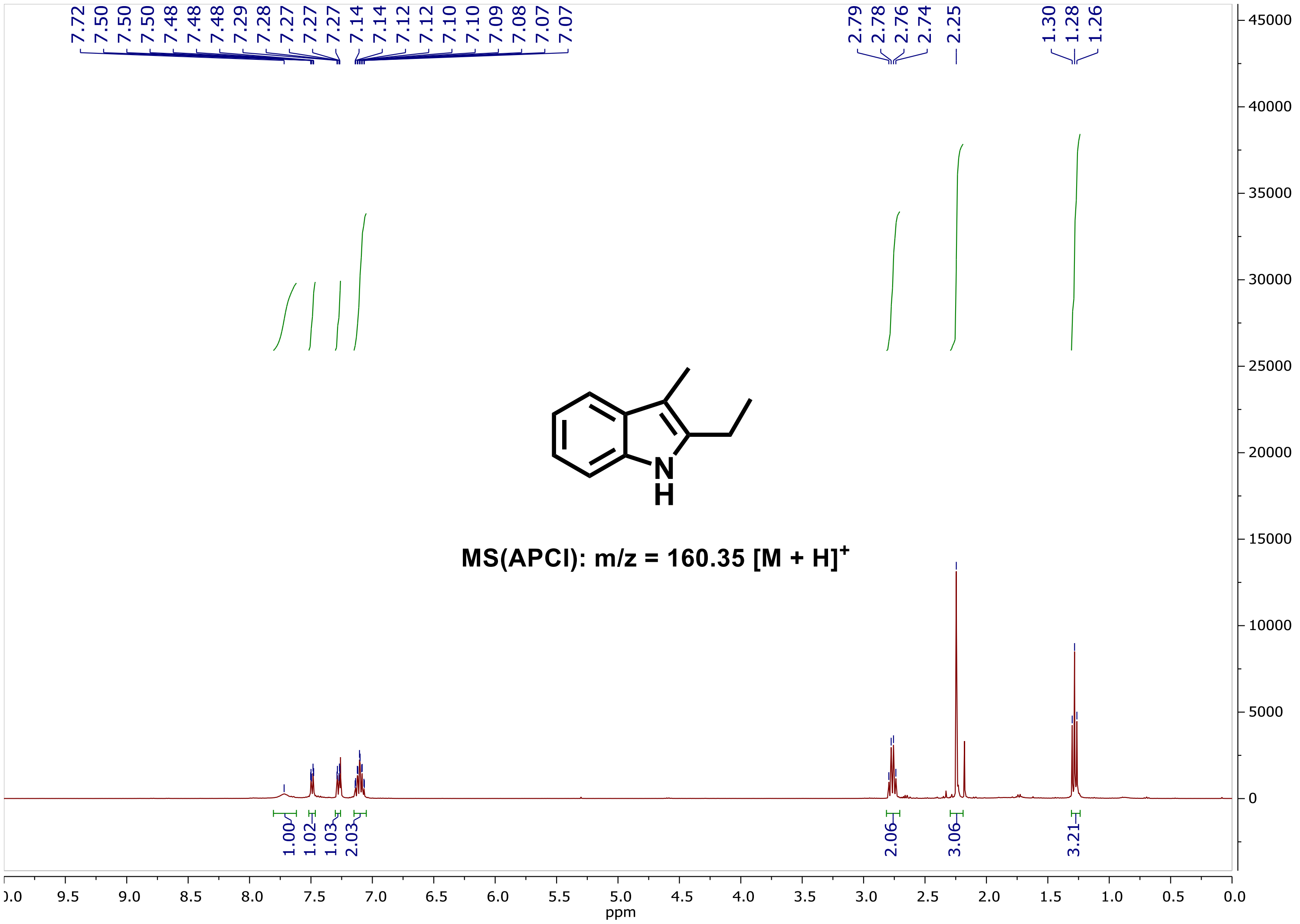

Fig. S13 ^{1}H NMR spectrum recorded for the product described in Table 2, Entry 7 ($CDCl_3$). Data were in accord with literature values.[3]

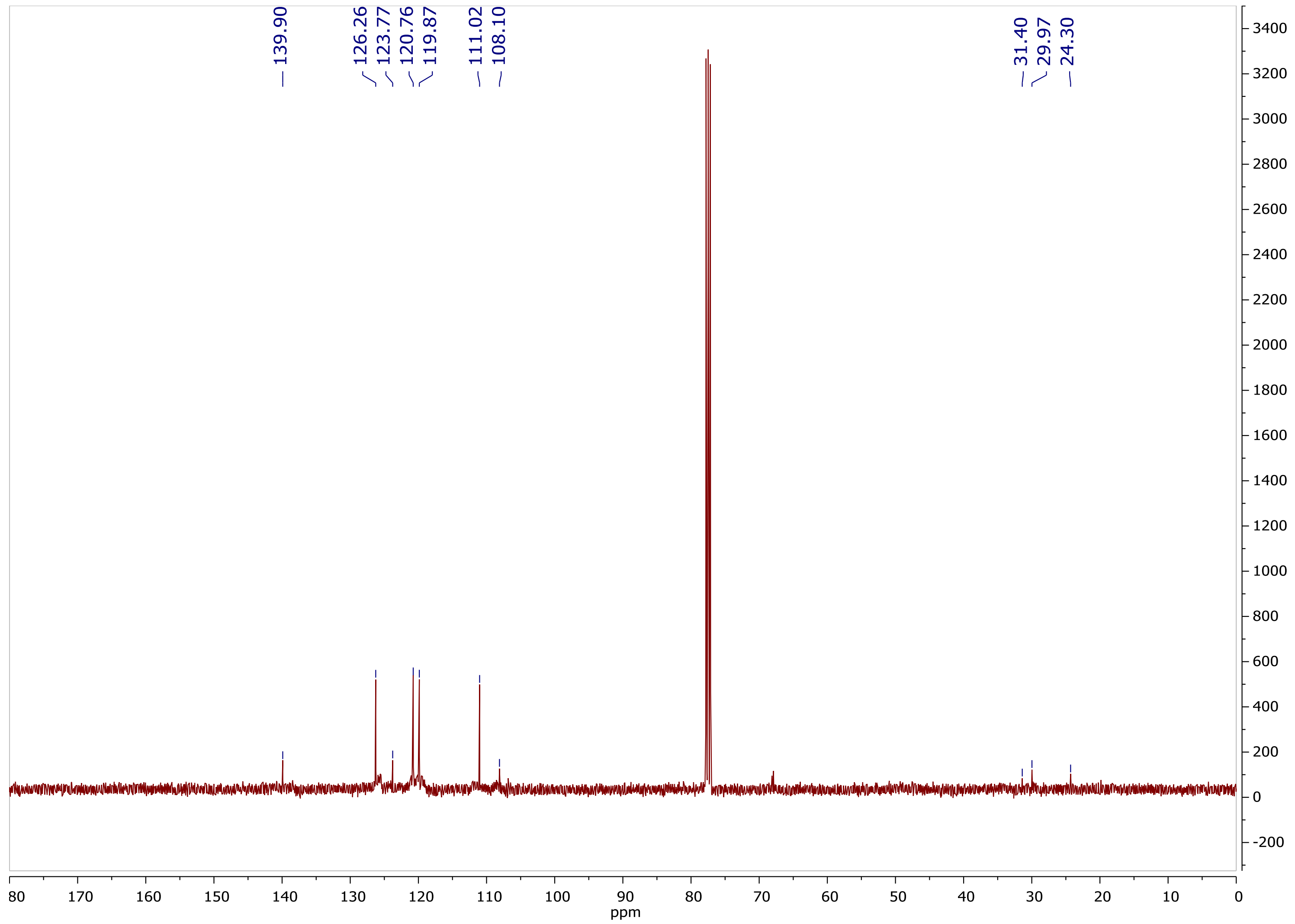

Fig. S14 ^{13}C NMR spectrum recorded for the product described in Table 2, Entry 7 ($CDCl_3$).

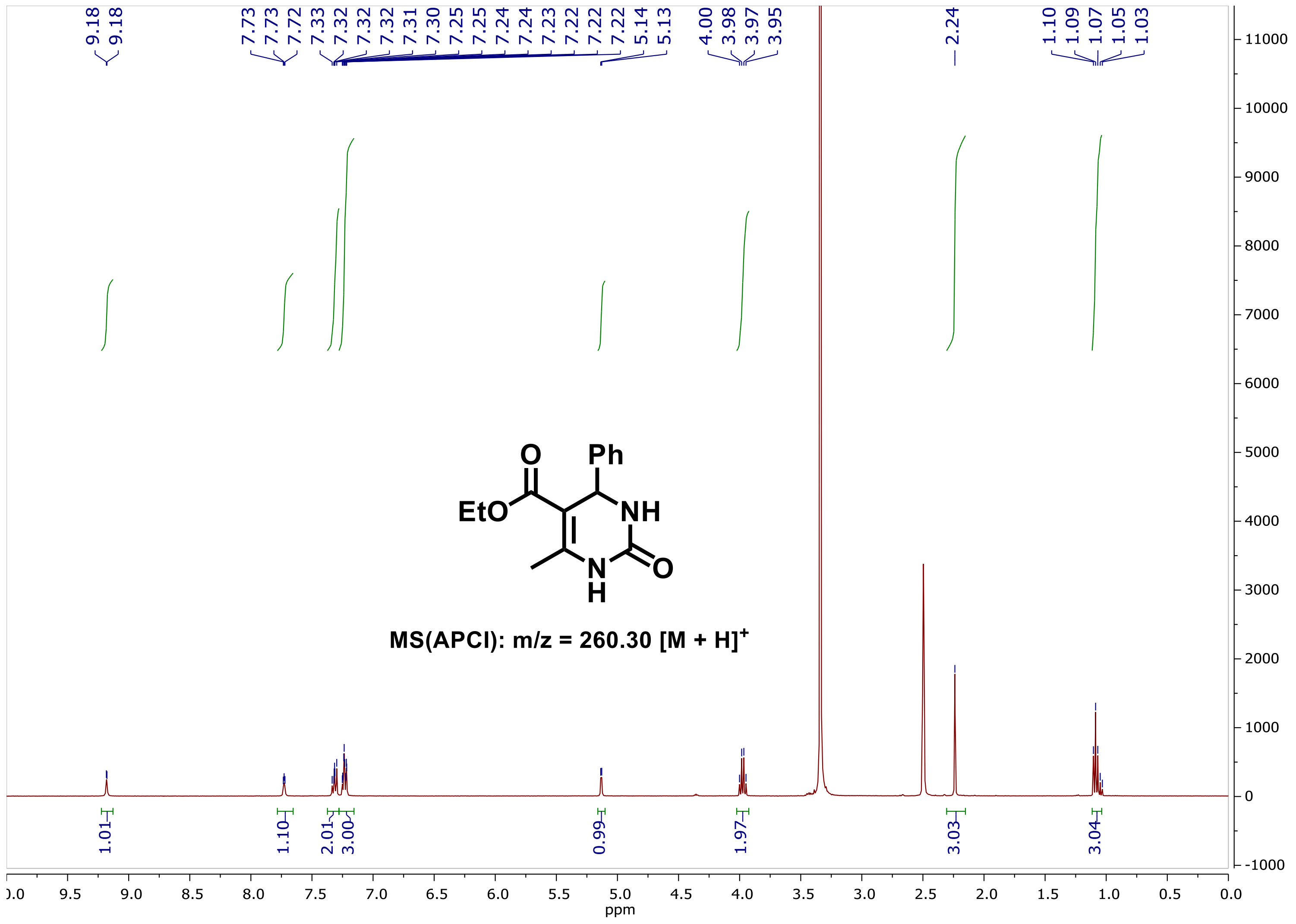

Fig. S15 ^{1}H NMR spectrum recorded for the product described in Table S4, Entry 1 (DMSO-d_6). Data were in accord with literature values.[4]

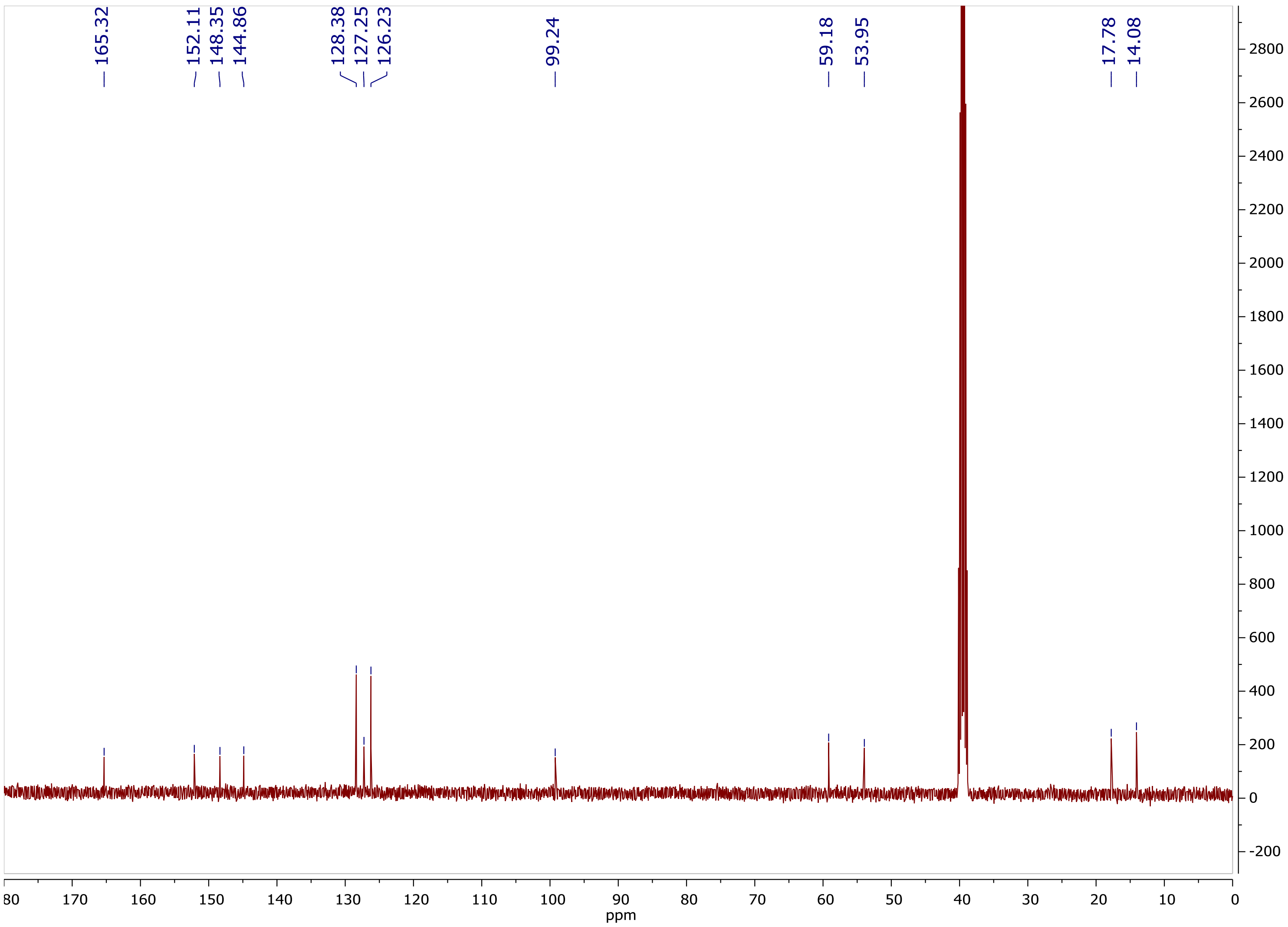

Fig. S16 ^{13}C NMR spectrum recorded for the product described in Table S4, Entry 1 (DMSO-d_6).

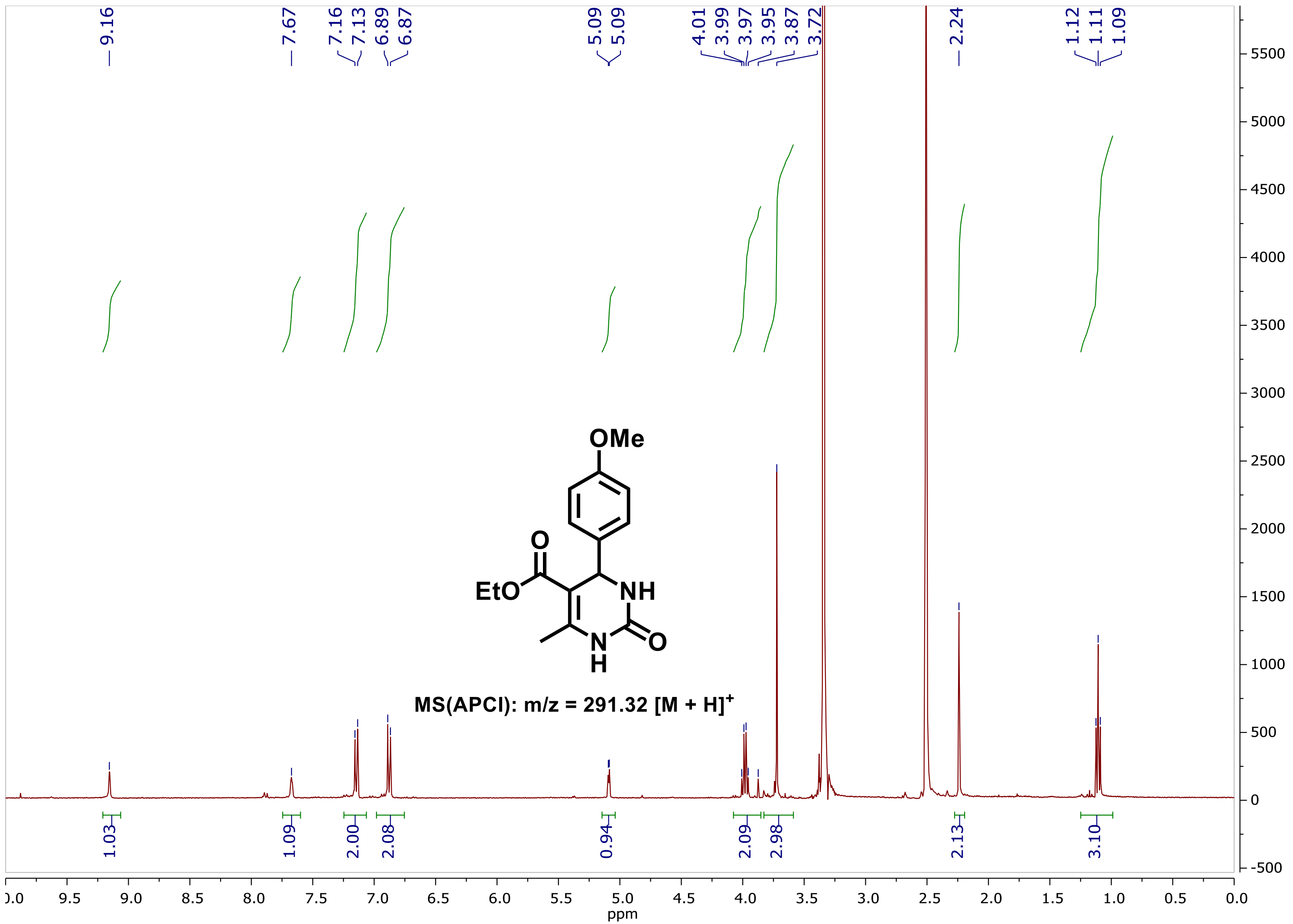

Fig. S17 ^{1}H NMR spectrum recorded for the product described in Table S4, Entry 2 (DMSO-d_6). Data were in accord with literature values.[4]

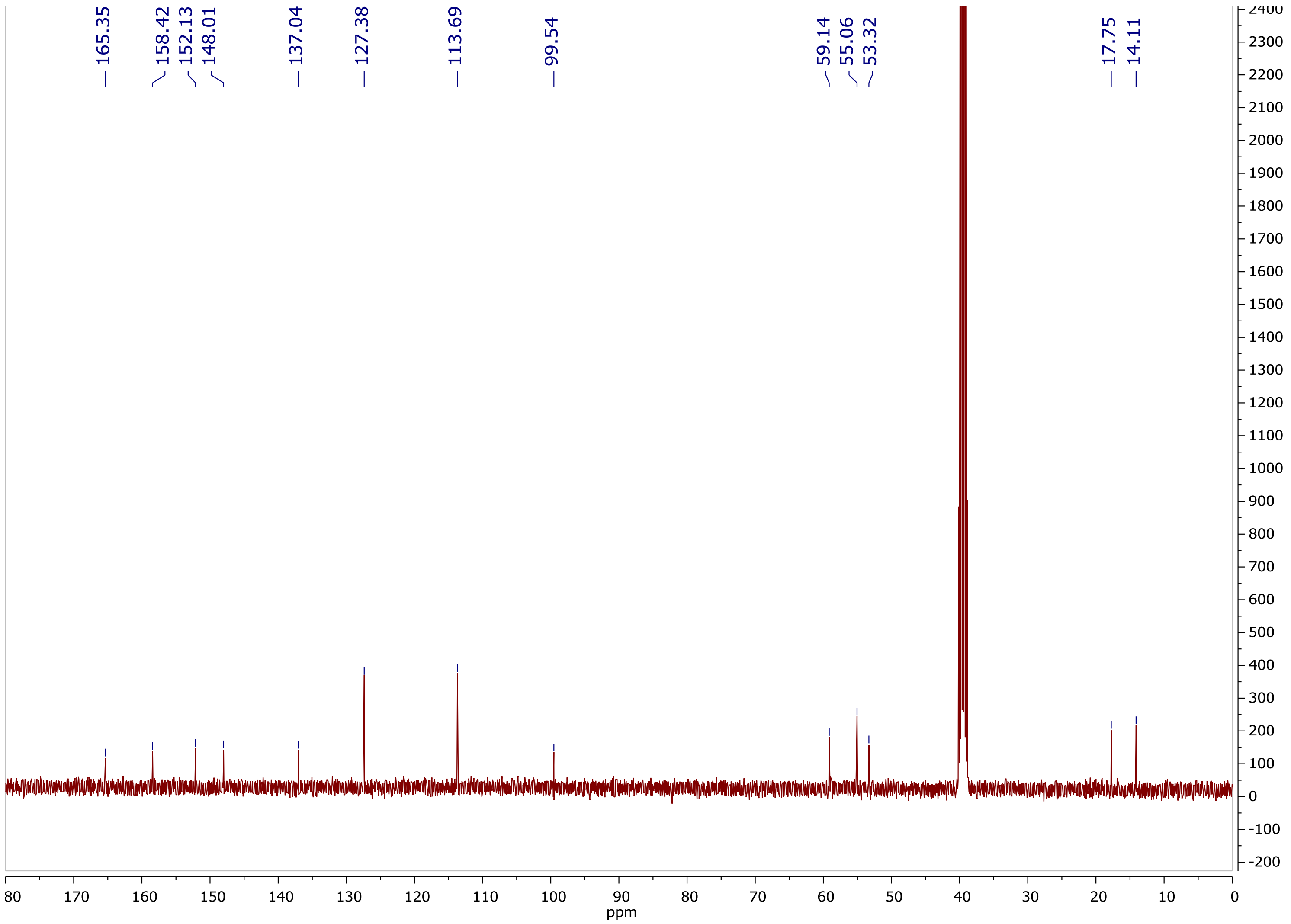

Fig. S18 ^{13}C NMR spectrum recorded for the product described in Table S4, Entry 2 (DMSO-d_6).

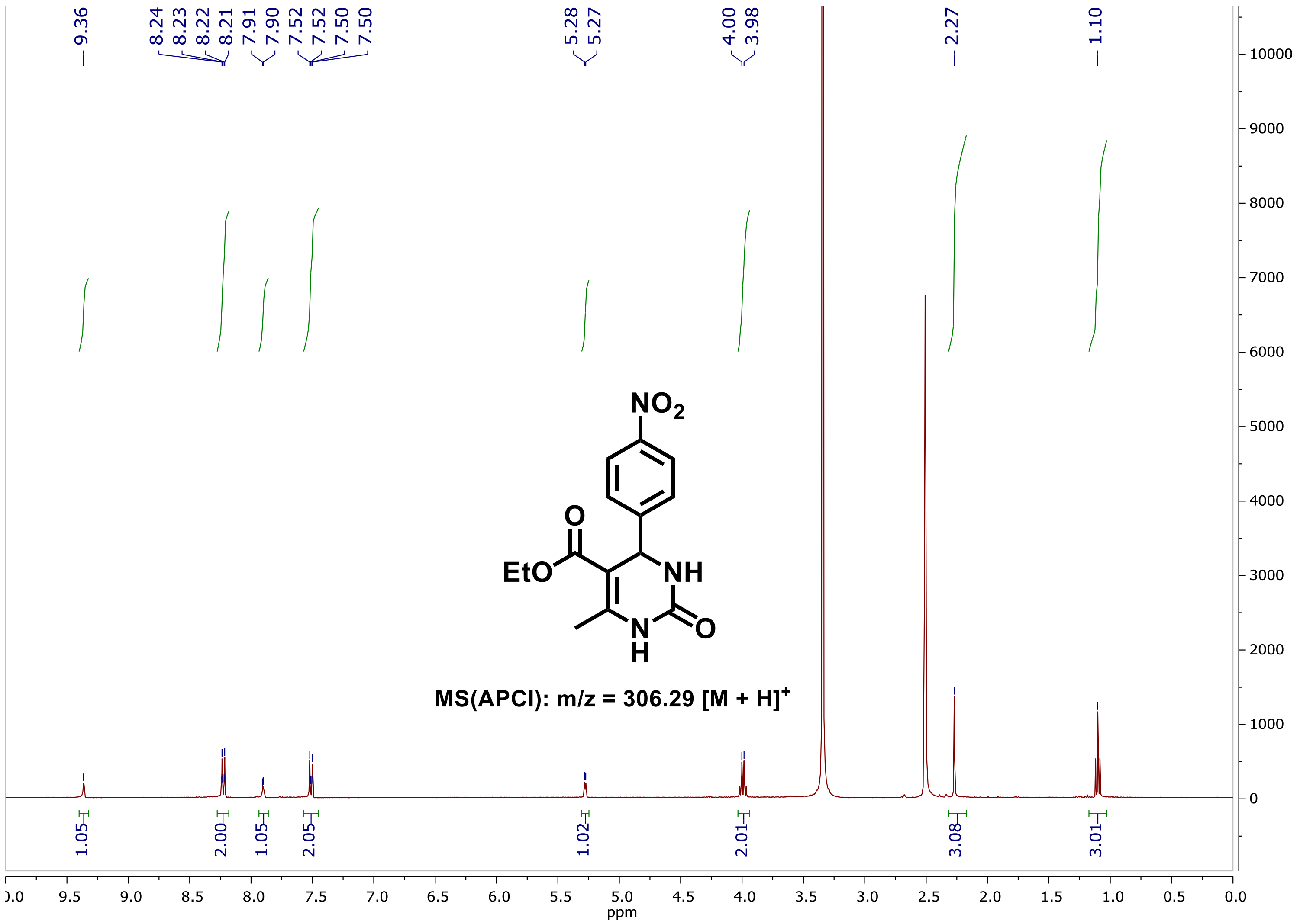

Fig. S19 ^{1}H NMR spectrum recorded for the product described in Table S4, Entry 3 (DMSO-d_6). Data were in accord with literature values.[4]

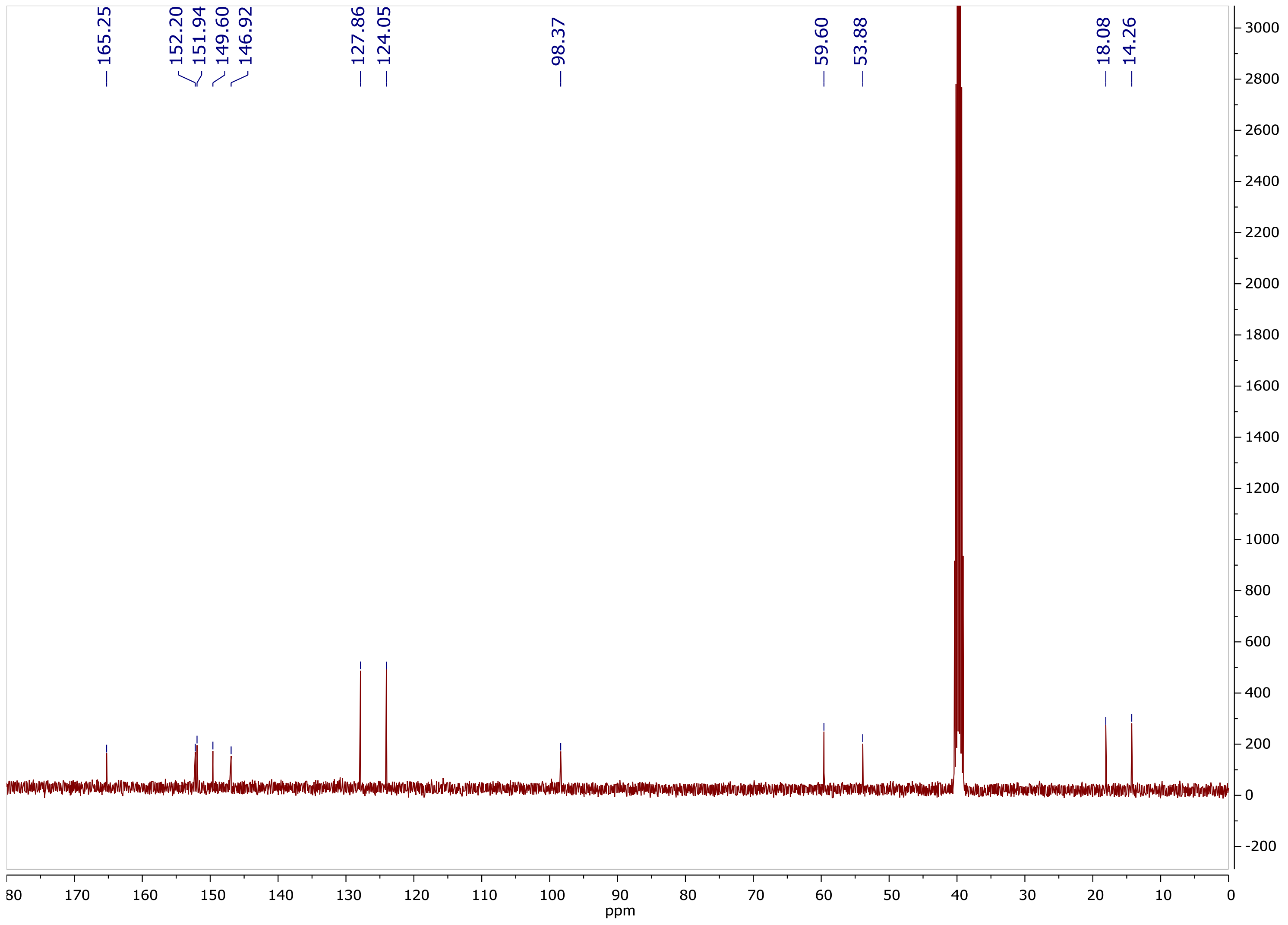

Fig. S20 ^{13}C NMR spectrum recorded for the product described in Table S4, Entry 3 (DMSO-d_6).

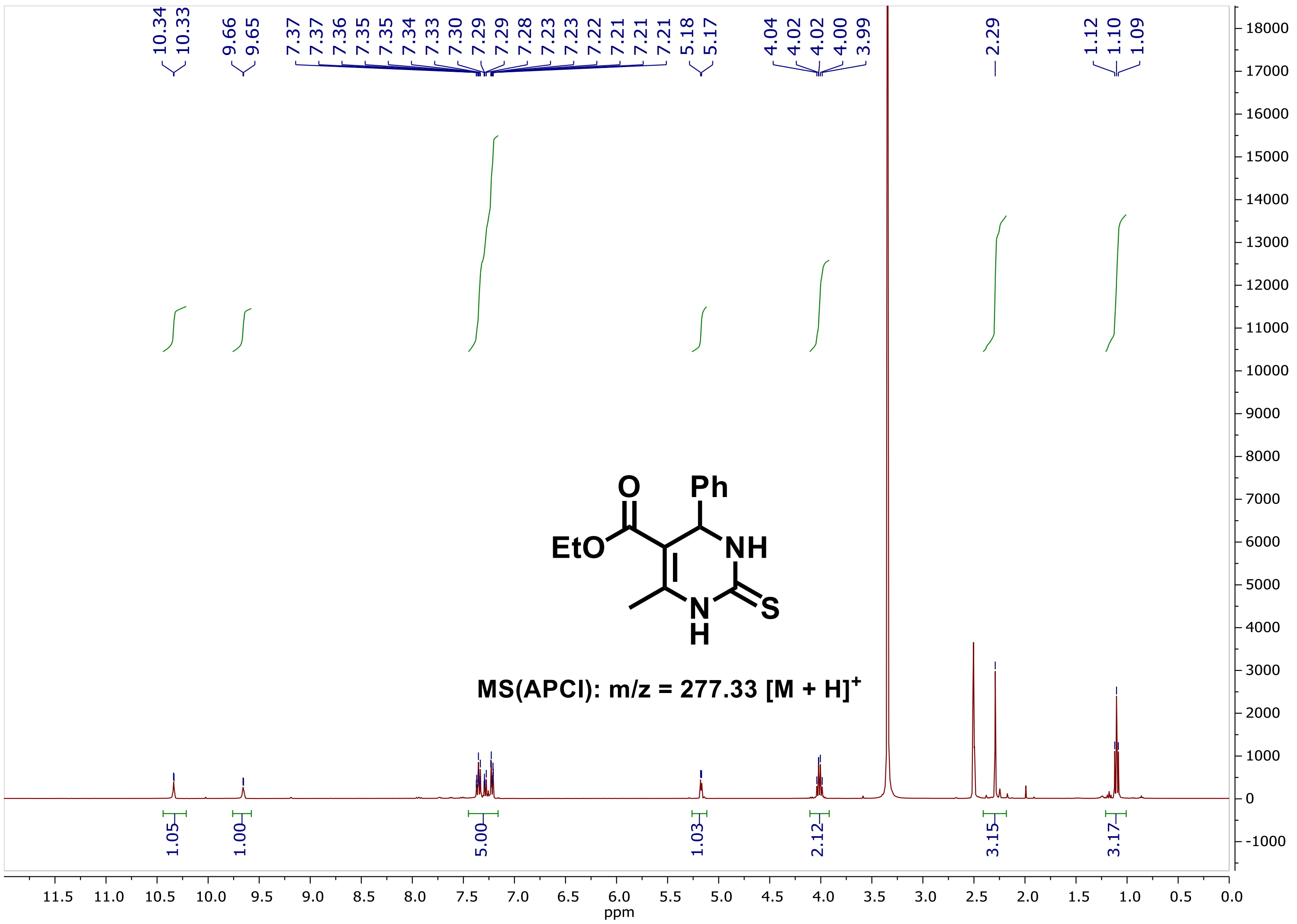

Fig. S21 ^{1}H NMR spectrum recorded for the product described in Table S4, Entry 4 (DMSO-d_6). Data were in accord with literature values.[5]

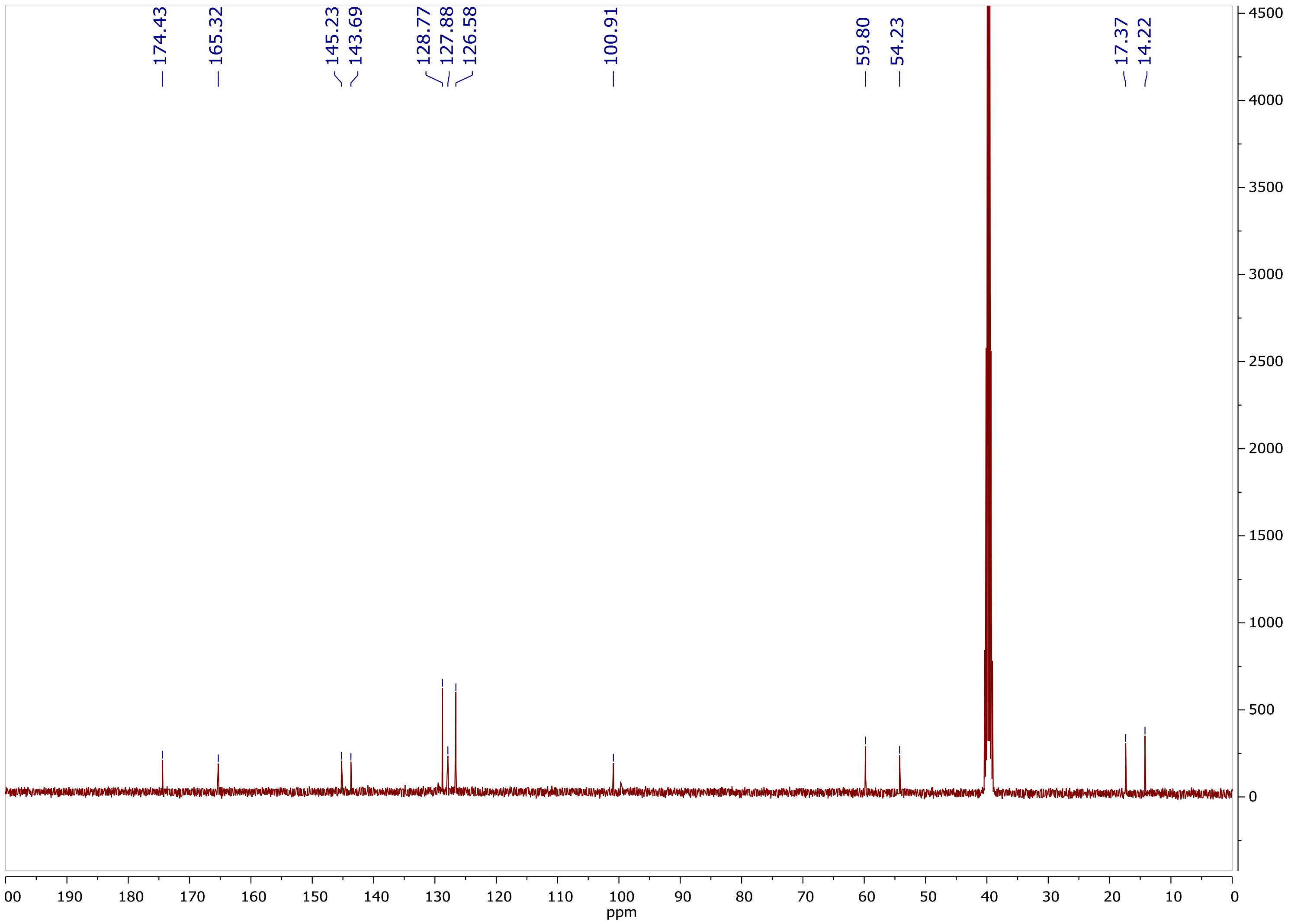

Fig. S22 ^{13}C NMR spectrum recorded for the product described in Table S4, Entry 4 (DMSO-d_6).

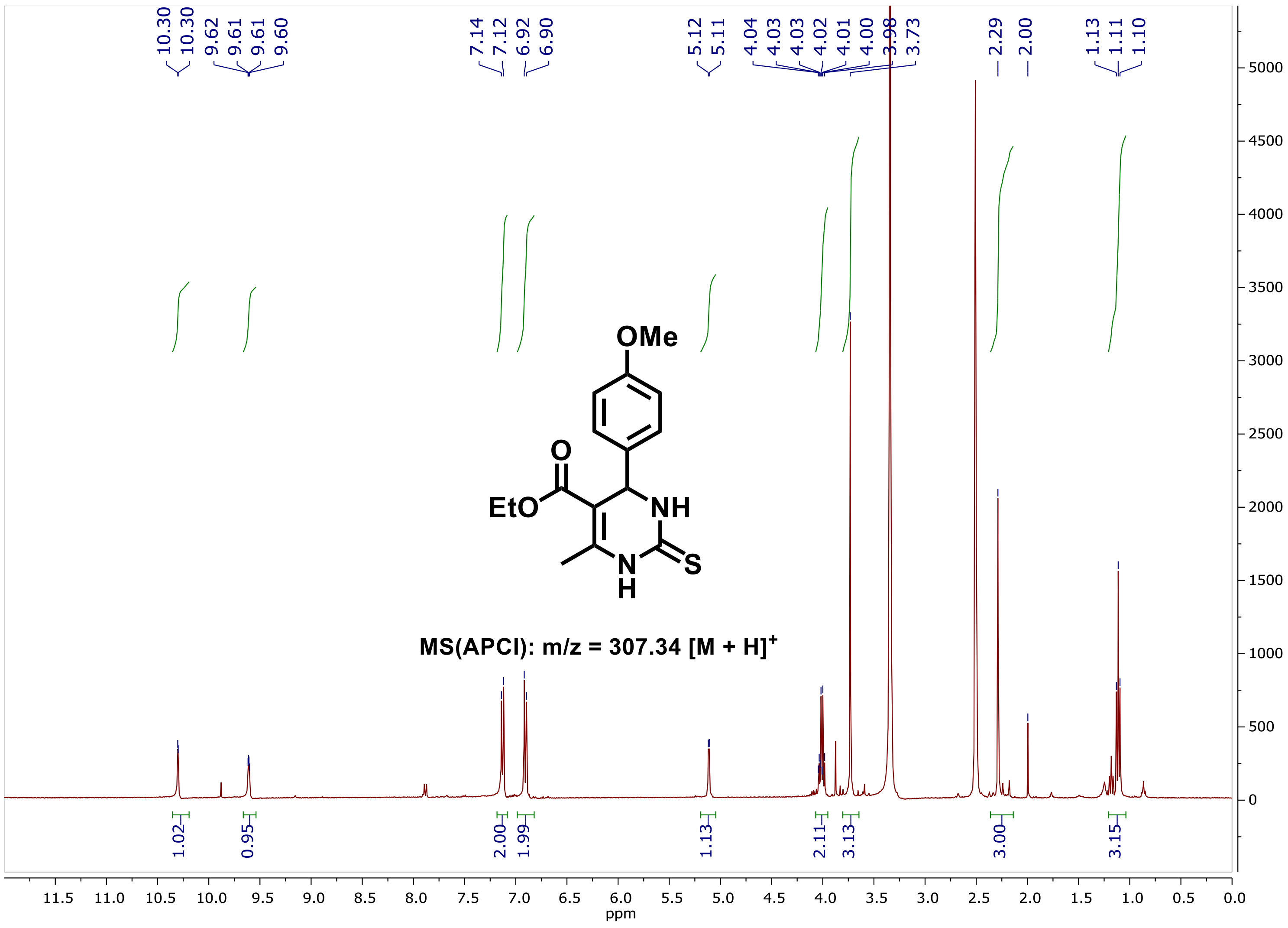

Fig. S23 ^{1}H NMR spectrum recorded for the product described in Table S4, Entry 5 (DMSO-d_6). Data were in accord with literature values.[5]

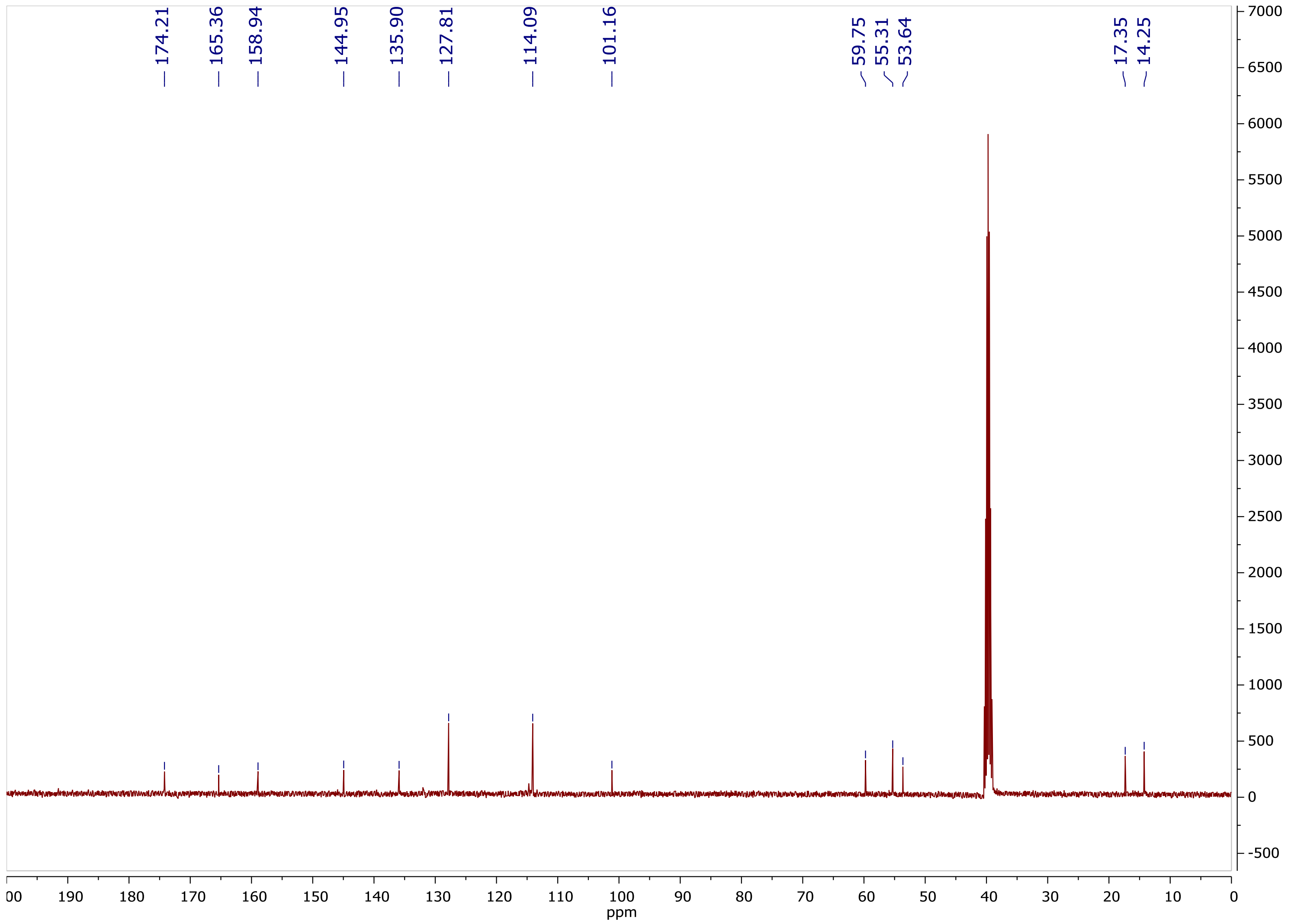

Fig. S24 ^{13}C NMR spectrum recorded for the product described in Table S4, Entry 5 (DMSO-d_6).

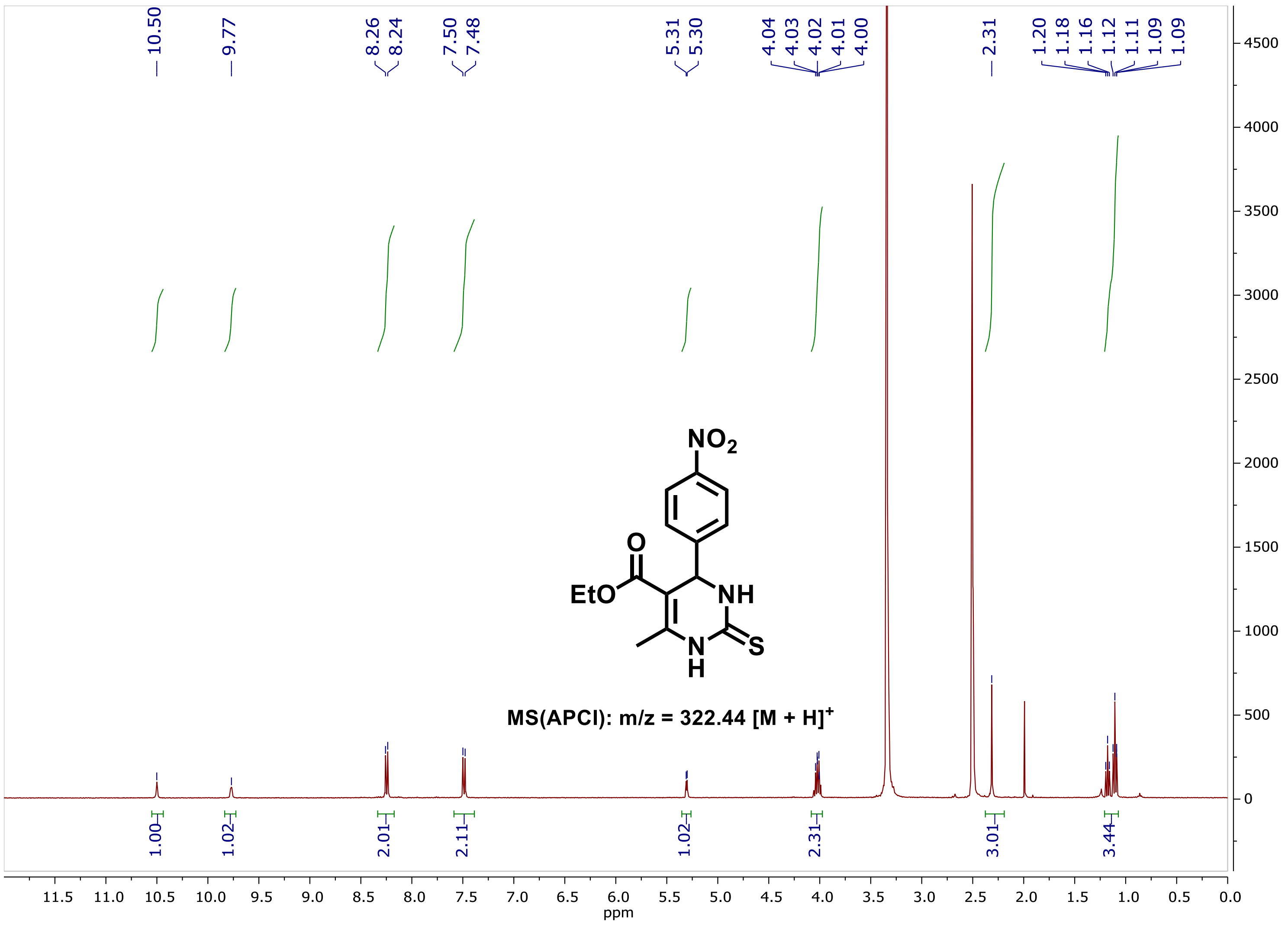

Fig. S25 ^{1}H NMR spectrum recorded for the product described in Table S4, Entry 6 (DMSO-d_6). Data were in accord with literature values.[6]

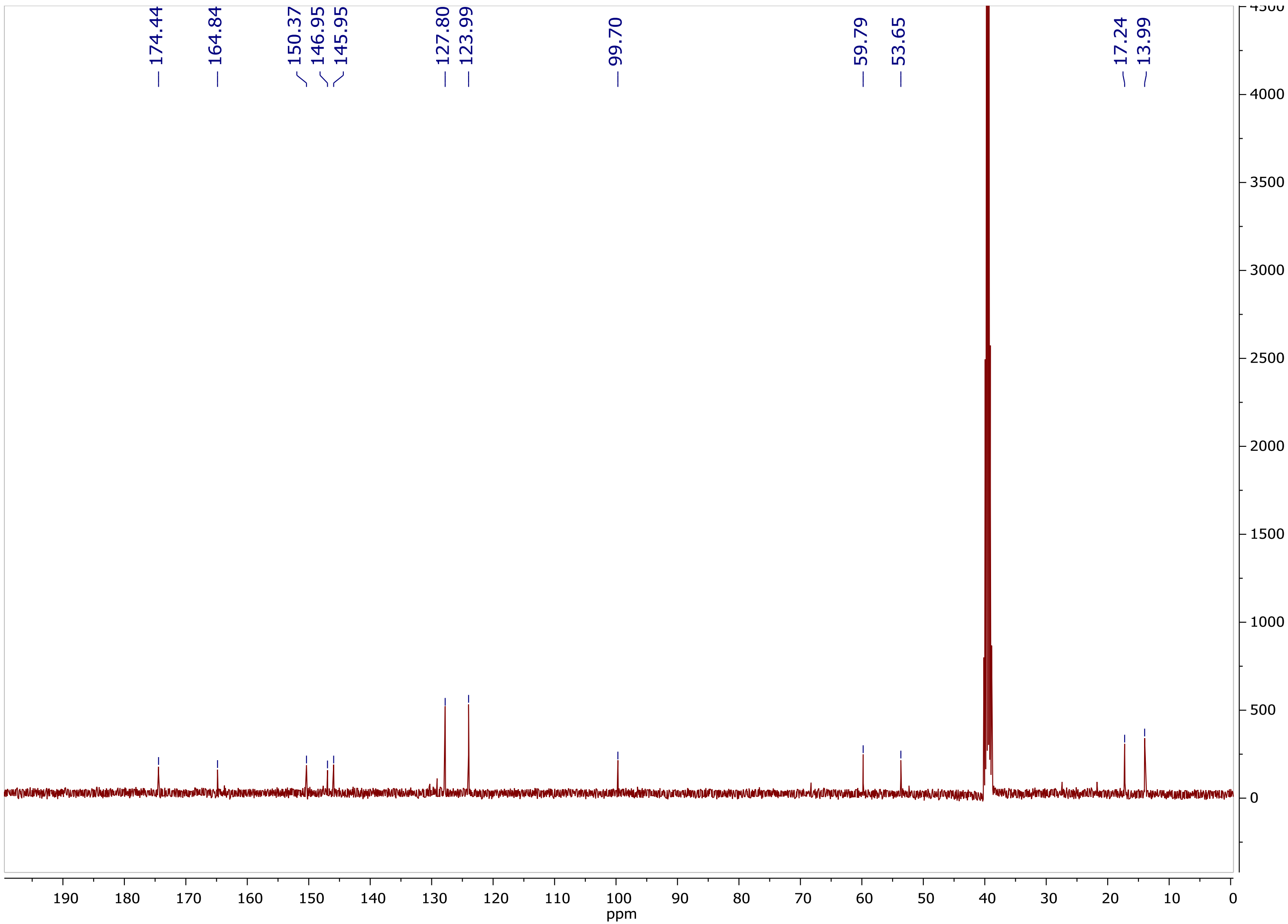

Fig. S26 ^{13}C NMR spectrum recorded for the product described in Table S4, Entry 6 (DMSO-d_6).

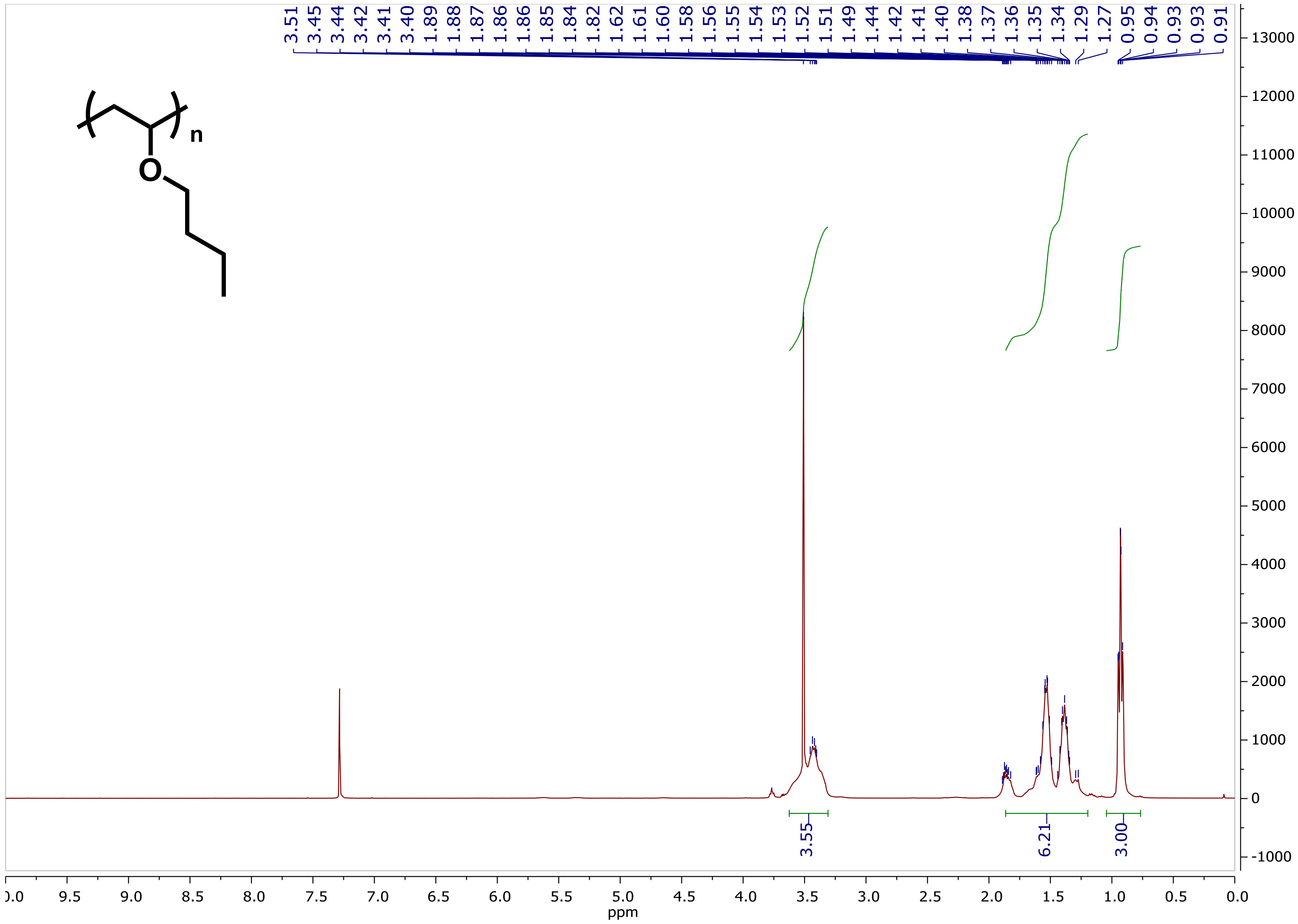

Fig. S27 ^{1}H NMR spectrum recorded for the product described in Table 4, Entry 1 ($CDCl_3$). Data were in accord with literature values.[7]

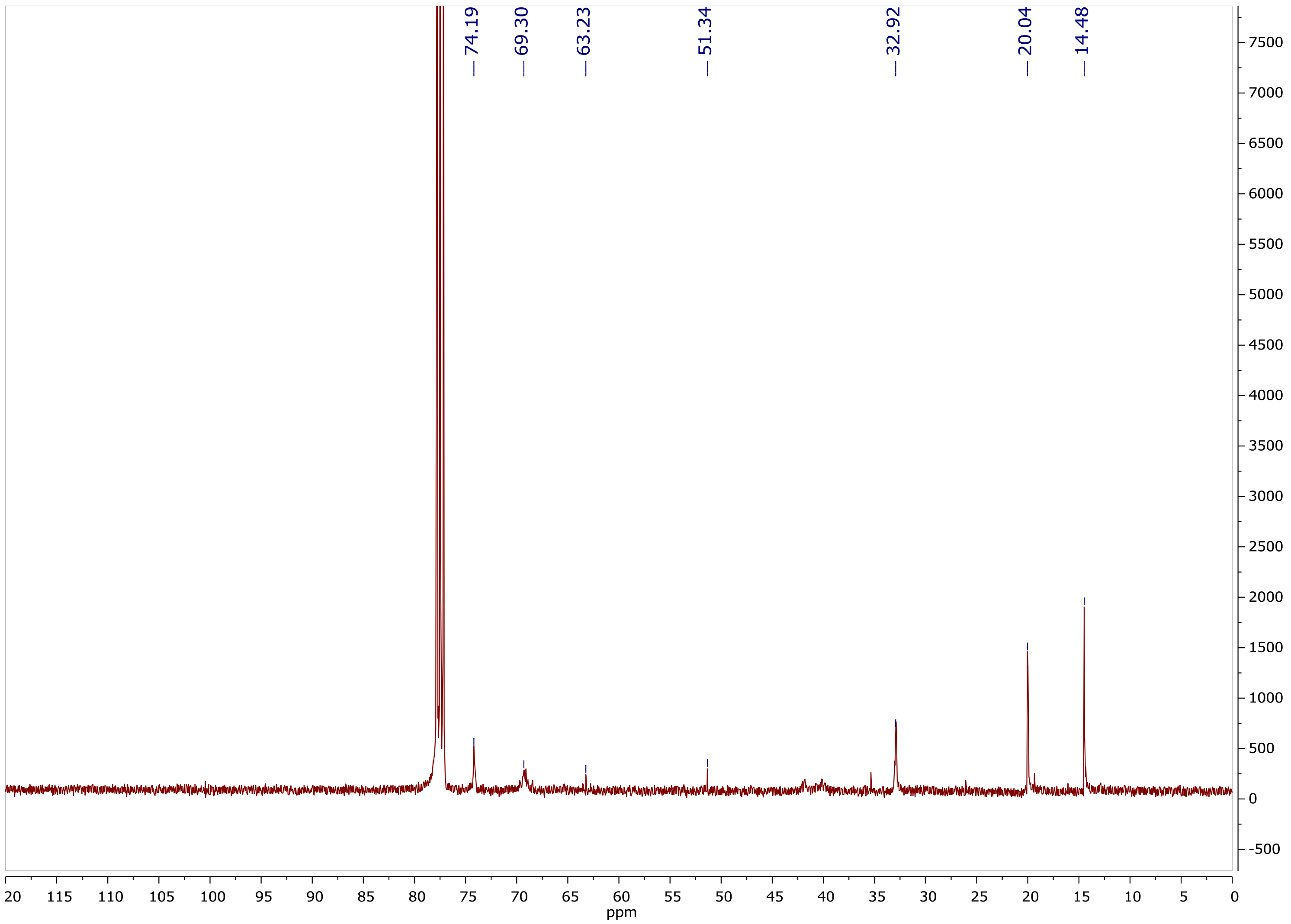

Fig. S28 ^{13}C NMR spectrum recorded for the product described in Table 4, Entry 1 ($CDCl_3$).

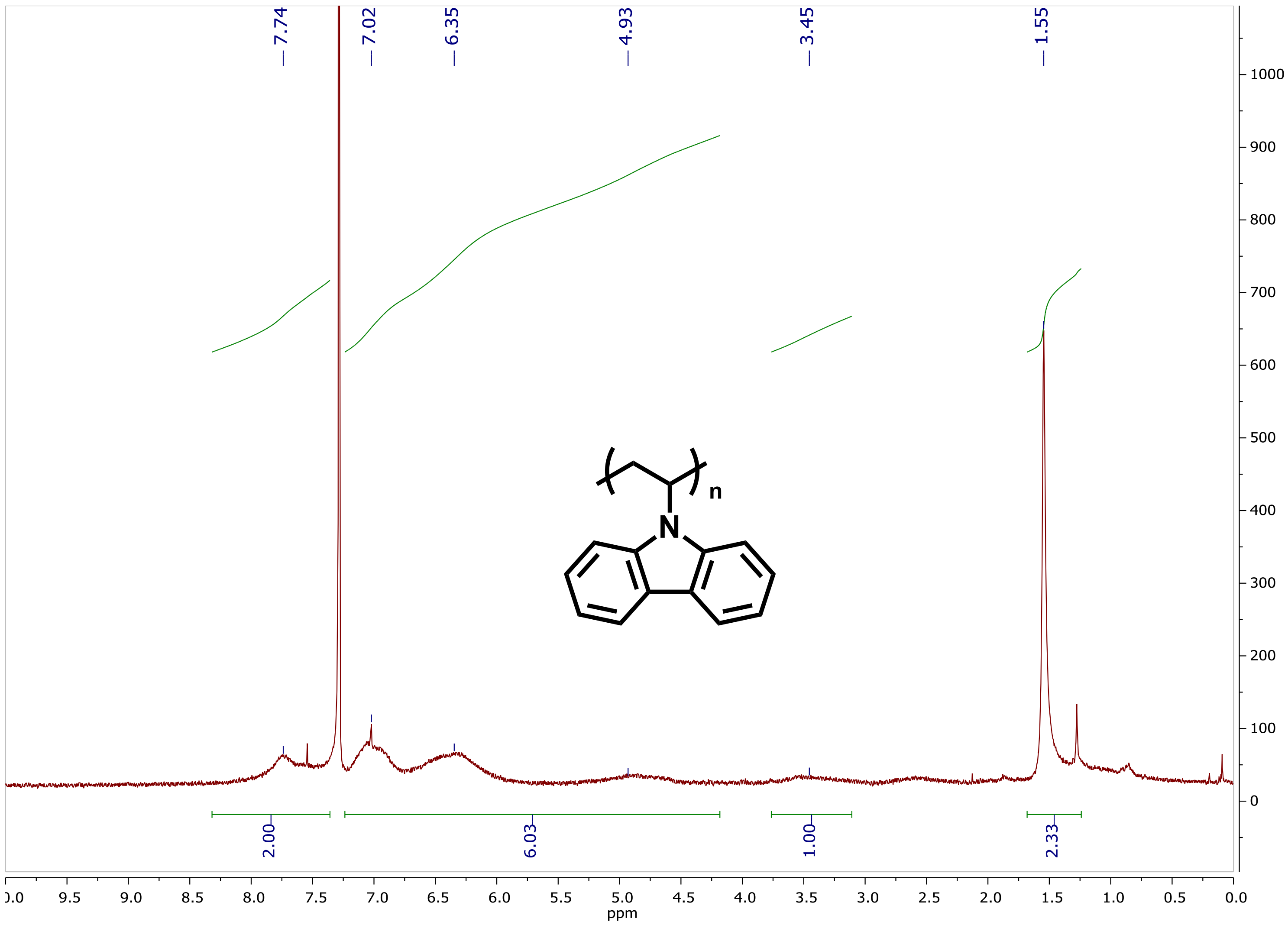

Fig. S29 ^{1}H NMR spectrum recorded for the product described in Table 4, Entry 2 ($CDCl_3$). Data were in accord with literature values.[8]

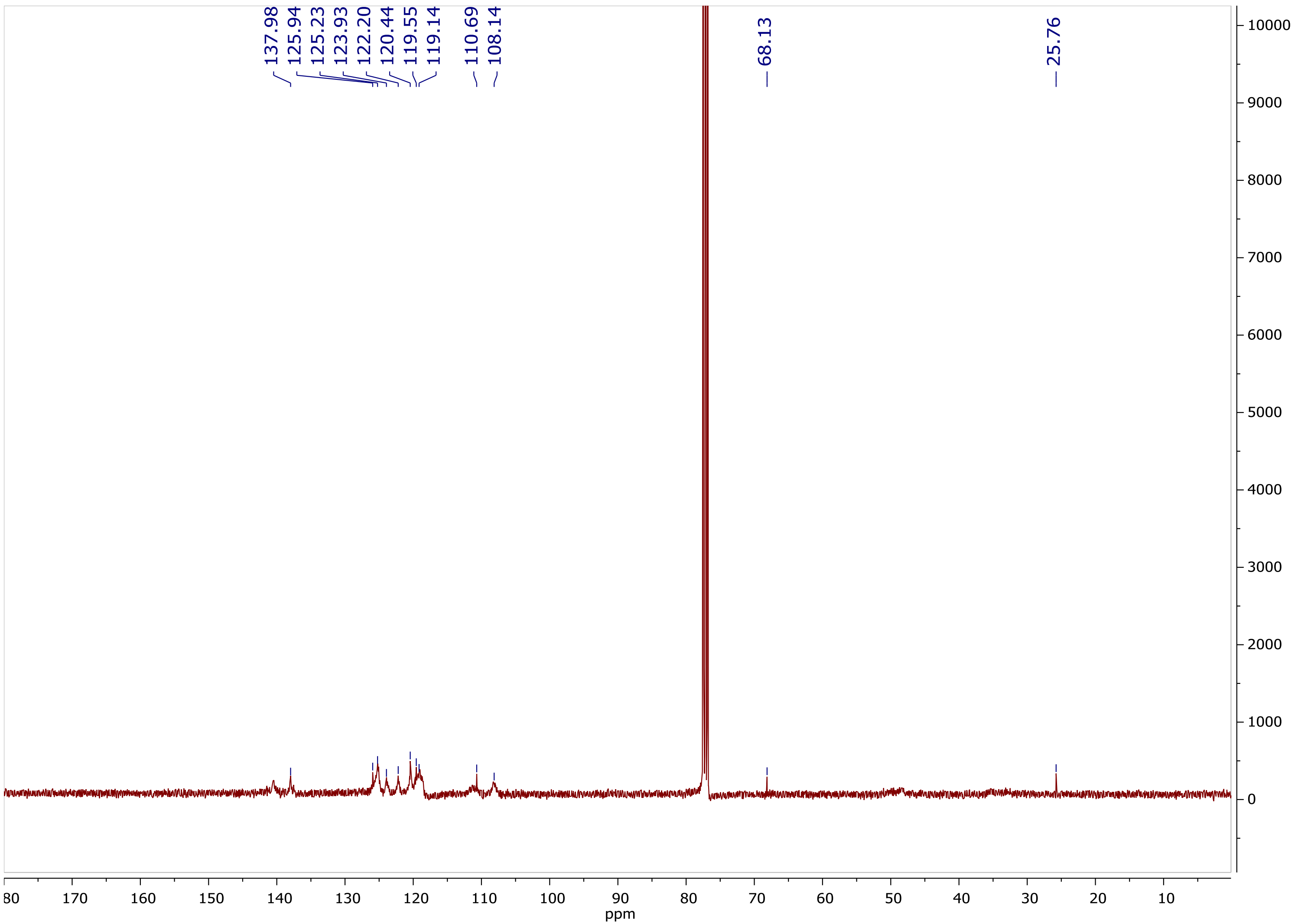

Fig. S30 ^{13}C NMR spectrum recorded for the product described in Table 4, Entry 2 ($CDCl_3$).

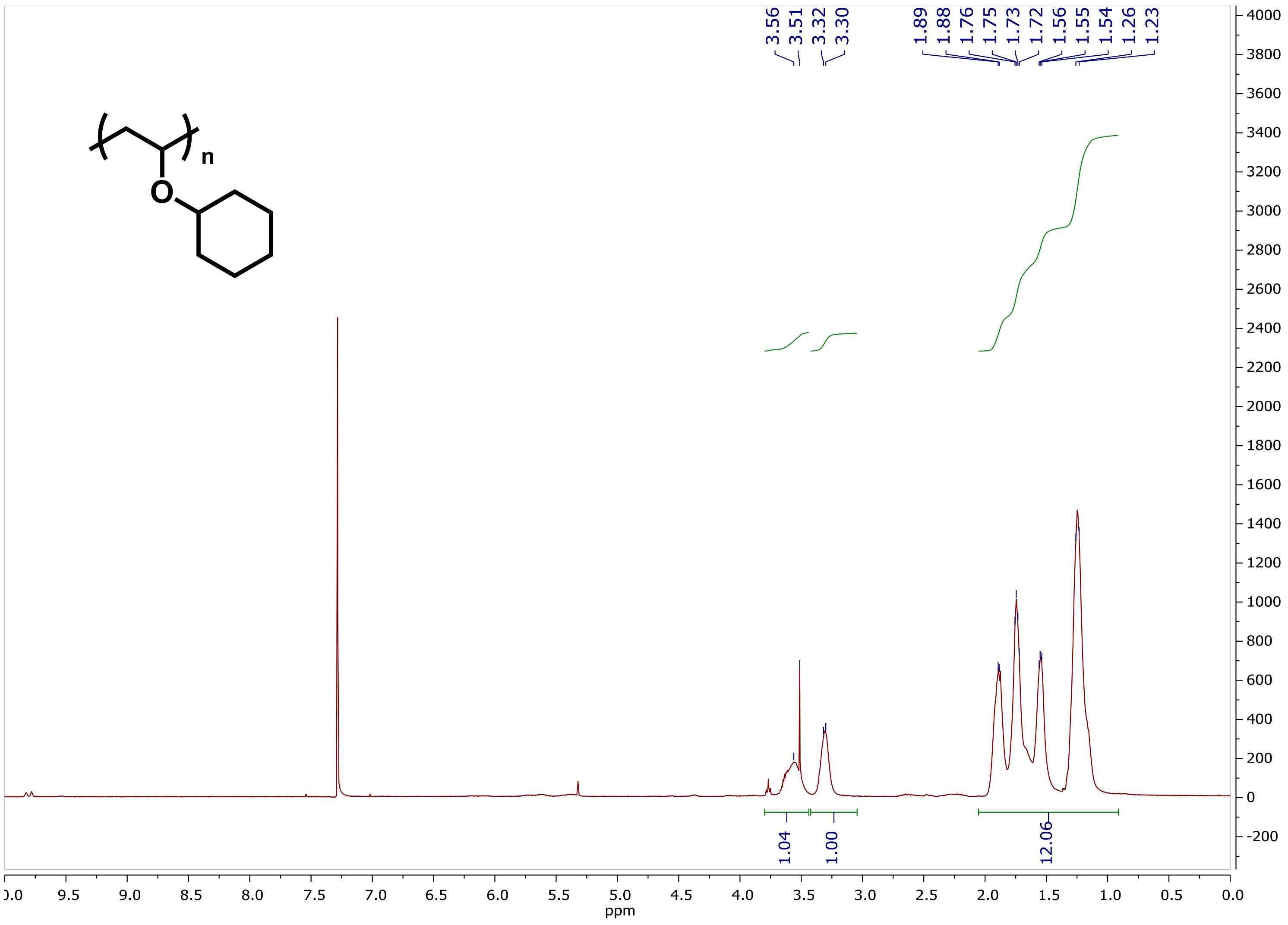

Fig. S31 ^{1}H NMR spectrum recorded for the product described in Table 4, Entry 3 ($CDCl_3$). Data were in accord with literature values.[9]

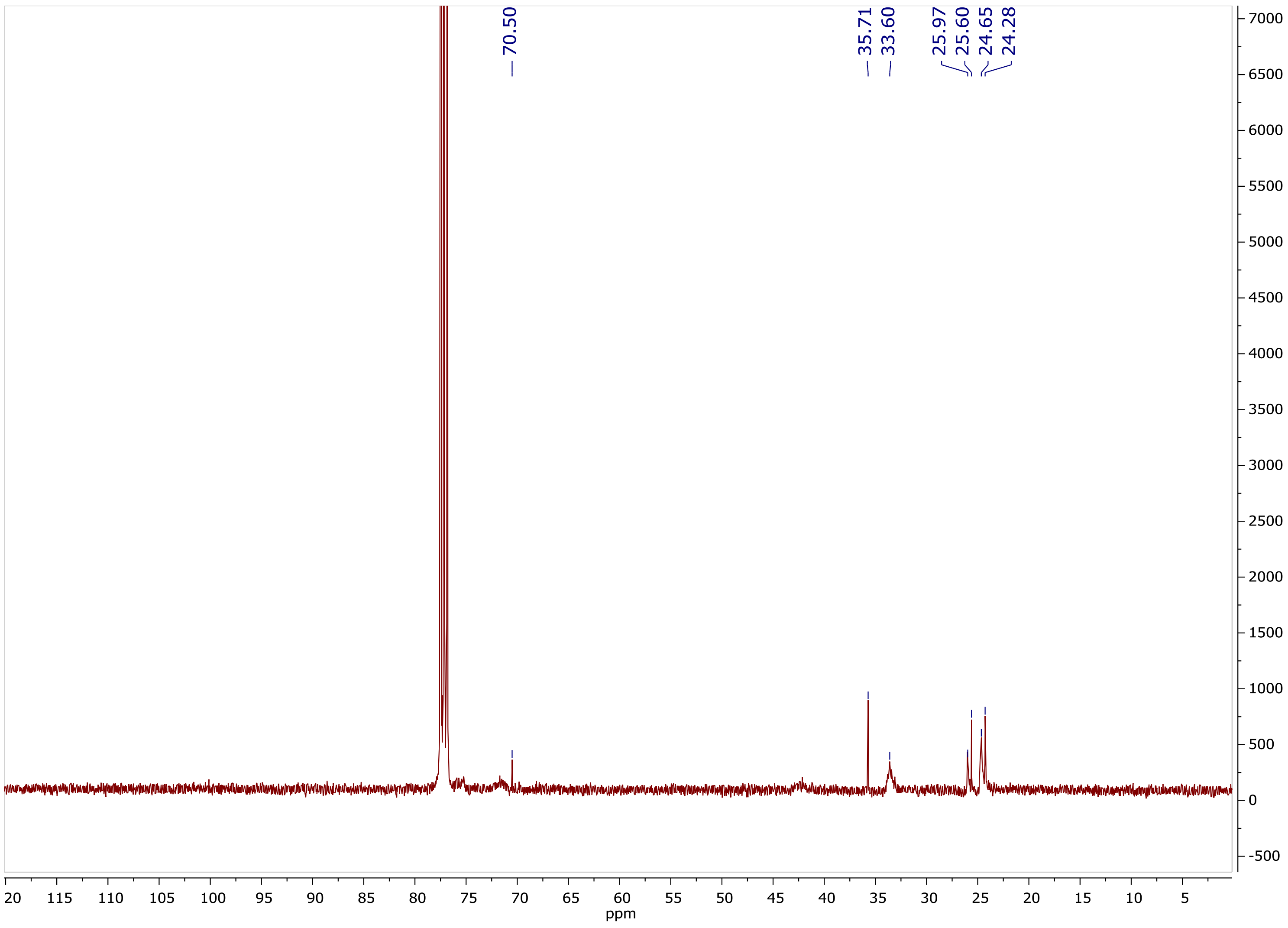

Fig. S32 ^{13}C NMR spectrum recorded for the product described in Table 4, Entry 3 ($CDCl_3$).

REFERENCES

1. B. Neises and W. Steglich, *Angew. Chem. Int. Ed. Engl.,* 1978, **17**, 522-524.

2. W. R. Collins, E. Schmois and T. M. Swager, *Chem. Commun.,* 2011, **47**, 8790-8792.

3. D. Q. Xu, J. Wu, S. P. Luo, J. X. Zhang, J. Y. Wu, X. H. Du and Z. Y. Xu, *Green Chem.,* 2009, **11**, 1239-1246.

4. E. Abbaspour-Gilandeh, S. C. Azimi and A. Mohammadi-Barkchai, *RSC Adv.,* 2014, **4**, 54854-54863.

5. S. R. Jetti, A. Upadhyaya, S. Jain, *Med. Chem. Res.*, 2014, **23**, 4356-4366

6. H. M. Savanur, R. G. Kalkhambkar, G. Aridoss, K. K. Laali, *Tetrahedron Lett.*, 2016, **57**, 3029-3035

7. H. Q. Guo, A. Kajiwara, Y. Morishima, M. Kamachi, W. Schnabel, *Polym. J.*, 1997, **29**, 446-449

8. D. K. Hazra, R. Chatterjee, *J. Mol. Struct.*, 2013, **1045**, 139-144

9. Y. Zhou, R. Faust, R. Richard, M. Schwarz, *Macromolecules*, 2005, **38**, 8183-8191
